# Regulatory Coordination of Photophysical, Photochemical, and Biochemical Reactions in the Photosynthesis of Land Plants

**DOI:** 10.1002/pld3.70080

**Published:** 2025-05-26

**Authors:** Lianhong Gu, Bernard Grodzinski, Jimei Han, Telesphore Marie, Yong‐Jiang Zhang, Yang C. Song, Ying Sun

**Affiliations:** ^1^ Environmental Sciences Division and Climate Change Science Institute Oak Ridge National Laboratory Oak Ridge Tennessee USA; ^2^ Department of Plant Agriculture University of Guelph Guelph Ontario Canada; ^3^ School of Integrative Plant Science Cornell University Ithaca New York USA; ^4^ School of Biology and Ecology University of Maine Orono Maine USA; ^5^ Department of Hydrology and Atmospheric Sciences University of Arizona Tucson Arizona USA

**Keywords:** electron transport regulation, granal thylakoid, photosynthesis modeling, plant energy use strategies, plant water use strategies

## Abstract

Balance among the sequential photophysical, photochemical, and biochemical reactions of photosynthesis is needed for converting fleeting energy in light to stable energy in chemical bonds. Any imbalance acts as either a bottleneck for limiting photosynthetic efficiency or an agent for inducing structural and functional damage to photosynthetic apparatus. Not only must each reaction be carefully regulated, but regulatory processes must also be coordinated across the reactions. However, regulations of different stages of photosynthesis have rarely been studied jointly. Non‐photochemical quenching (*NPQ*) and stomatal conductance (*g*_s_) are key regulators of photophysical and biochemical reactions, respectively. Existing evidence suggests that the redox state of plastoquinone regulates *g*_s_ and that the photochemical reactions are partially regulated by the ultrastructural dynamics of thylakoids induced by osmotic water fluxes in chloroplasts of land plants. To examine how these regulations are coordinated and feedback to each other, we simultaneously measured *NPQ* and *g*_*s*_ and inferred the redox state of plastoquinone and the light‐induced thylakoid swelling/shrinking on numerous C_3_ and C_4_ species. For all species measured, *NPQ* and *g*_*s*_ covary with the redox states of the electron transport chain, particularly plastoquinone, and increase as thylakoid swelling is inferred. *NPQ* has the maximal sensitivity at the light intensity at which thylakoid is inferred to be fully swollen. Our findings suggest that plant energy and water use strategies are intimately linked by evolution, and studying the regulations of different photosynthetic stages as a whole can lead to new insights of the functioning of photosynthetic machinery in dynamic environments.

## 1 Introduction

Photosynthesis is conducted through interconnected events that can be broadly divided into photophysical reactions, photochemical reactions, and biochemical reactions. These three groups of reactions take place serially and in separate domains, are driven by different energy forms, proceed at vastly contrasting speeds, and follow different laws (Kamen 1963; Gu et al. 2023). During the photophysical reactions, light energy is harvested by chlorophylls and carotenoids and transformed into excitation energy, which is then dissipated along pathways of photochemistry, regulated and unregulated heat release, and fluorescence emission. During the photochemical reactions, excitons funneled to the reaction centers are used to drive electrons down the electron transport chain (ETC) and proton translocation from the stroma to the lumen, resulting in the release of oxygen and the syntheses of electron transport end products such as NADPH, ATP, and intermediate reductants. During the biochemical reactions, NADPH and ATP formed at the end of the photochemical reactions are used to support the assimilation of CO_2_, which diffuses from the ambient air to the intercellular airspace through stomata, whereas intermediate reductants may be used to support other assimilations such as nitrate reduction. A fundamental issue for plant photosynthesis is how to keep all these disparate reactions in unison in natural environments that can fluctuate unpredictably.

Plants have evolved delicate regulatory mechanisms to ensure that supply (input)–demand (output) balances are achieved among the three stages of reactions, which is essential for avoiding production of reactive oxygen species, protecting sensitive tissues and organs, and sustaining photosynthesis in fluctuating environments. These mechanisms may be triggered by processes in one stage but act on targets in another. For convenience, they can be discussed based on the order of targets in the photophysical, photochemical, and biochemical reactions. Non‐photochemical quenching (NPQ), which dissipates the excess absorbed light energy as harmless heat, is a major mechanism for regulating the photophysical reactions (Ruban 2016). Although NPQ itself is regulated by the acidity in the lumen, its effect is to decrease the population of chlorophylls in the singlet excited states. This decrease minimizes the population of chlorophylls in the dangerous triplet excited states, which are produced from the singlet states via intersystem crossing and can form reactive radicals that photo‐oxidatively damage cells. Because this quenching action takes place in the light‐harvesting antenna complexes before photochemistry is initiated (Nicol et al. 2019; Bassi and Dall'Osto 2021), its protective role is maximized. Other regulation mechanisms for the photophysical reactions include state transition and chloroplast avoidance movement. State transition is a process of repositioning the mobile light‐harvesting complex II (LHCII) to rebalance energy absorption and allocation between photosystem II (PSII) and photosystem I (PSI) (Minagawa 2011). It is activated by the redox state of plastoquinone (PQ) to adjust the relative proportions of whole‐chain linear electron transport from PSII to PSI and the cyclic electron transport around PSI and therefore the production ratio of NADPH to ATP to satisfy the varying demands of the biochemical reactions in response to environmental variations (Kramer and Evans 2011). Under low sunlight, chloroplasts are typically located right under the cell surface for maximal light interception. Under strong light, however, they can move to the side walls of cells to minimize light exposure. Just like NPQ, this avoidance movement also plays a protective role to plants by reducing photodamage (Kasahara et al. 2002; Cazzaniga et al. 2013).

The long path between PSII and PSI, which are spatially segregated with PSII mostly in grana stacks and PSI in stromal lamellae and connected with electron carriers, offers different mechanisms to regulate the photochemistry. The rate‐limiting step along the ETC is believed to be the oxidation of plastoquinol by the cytochrome b_6_f complex (Cyt) (Laisk et al. 2005), which has been shown to be sensitive to the pH value of the lumen (Rochaix 2011). Linear and cyclic electron transports intersect at Cyt, which makes Cyt a prime target for photosynthetic controls (Foyer et al. 2012). Because both NPQ and photosynthetic controls are affected by lumen acidity, coordination between these two regulations is expected. It has been suggested that the diffusion of the electron carriers PQ and plastocyanin (PC) can be restricted by macromolecular crowding in the thylakoids and light‐induced thylakoid swelling could relieve this restriction (Kirchhoff et al. 2000, 2011; Kirchhoff et al. 2017; Höhner et al. 2020). Gu et al. (2022) put forward a theory to systematically explain how the environmentally inducible thylakoid ultrastructural dynamics might regulate the electron transport from PSII to PSI in land plants. Central to the theory are grana stacks, whose pervasiveness is unique to land plants. It proposes that grana stacks, like bellows of an accordion, increase the degree of ultrastructural control on redox reactions and diffusion of PQ and PC through thylakoid swelling/shrinking induced by osmotic water fluxes. Motivated by previous experimental observations and the theory, Gu et al. (2023) developed a redox reaction–based photochemical model. They showed that the light‐induced thylakoid swelling/shrinking must be explicitly represented for the model to successfully fit the observed relationships between the whole‐chain electron transport rate and the fraction of open PSII reaction centers across a large group of species and wide range of environmental conditions.

Although a great deal has been known about the regulations of the biochemical reactions of photosynthesis that have been successfully modeled for decades with the FvCB biochemical model (Farquhar et al. 1980), mechanisms of these regulations are interwoven and complex and remain to be thoroughly clarified (Lawson and Matthews 2020). Within the FvCB framework, the biochemical reactions are limited by either light availability, which limits electron transport for RuBP regeneration, or CO_2_ supply, which limits Rubisco activities. CO_2_ supply is controlled by the turgor pressure of guard cells whose swelling/shrinking due to osmotic water fluxes determines the aperture and therefore conductance (*g*_s_) of stomata. Guard cell turgor pressure and therefore *g*_s_ respond to a wide range of biotic and abiotic cues, allowing CO_2_ exchange to be maximized, whereas water loss to be minimized for a given set of environmental conditions (Cowan and Farquhar 1977; Medlyn et al. 2011). The maximum *g*_s_ is determined by leaf anatomy such as stomatal density, maximum stomatal aperture, and stomatal pore depth (Lawson and Matthews 2020). But the actual *g*_s_ depends on dynamic guard cell responses to environmental conditions such as light intensity, CO_2_ concentration, temperature, and humidity. Of particular interest to the present study is the *g*_s_ response to light, which consists of at least two mechanisms: blue and red light responses (Lawson and Matthews 2020). Blue light activates phototropins in guard cells to open stomata. This response is more like a switching effect rather than a dose response and saturates at light levels too low for CO_2_ assimilation. In contrast to the blue light response, red light produces a dose response from *g*_s_, which saturates at light intensities similar to those for photosynthesis. The mechanism of *g*_s_ red light response is still under active investigation. Experiments have demonstrated that when electron flow to PQ is impaired, *g*_s_ becomes insensitive to red light (Kuiper 1964; Dwyer et al. 2012); in contrast, when electron flow out of PQ is impaired, *g*_s_ remains sensitive to red light (Anderson et al. 1997). These findings, together with a synthesis of other experimental evidence, led Busch (2014) to suggest that a signal from the reduction level of the PQ pool mediates *g*_s_ red light response. A PQ‐mediated red light response of *g*_s_ is potentially advantageous for plant regulations of photosynthesis as the redox state of PQ already controls state transition and the NADPH to ATP synthesis ratio whose adjustments are needed for the photosynthetic machinery to properly respond to variations in environmental conditions (Kramer and Evans 2011). Consistent with the suggestion of Busch (2014), Głowacka et al. (2018) used the fraction of open PSII reaction centers (*q*_L_) as a proxy of the redox state of PQ and found that there was a tight correlation between *g*_s_ and *q*_L_ for tobacco plants ( *Nicotiana tabacum* ). Built upon these studies, Kromdijk et al. (2019) introduced a *q*_L_‐based *g*_s_ model with a form analogous to the widely used empirical models that were based on correlations between *g*_s_ and net CO_2_ assimilation rate (*A*_n_; Ball et al. 1986; Medlyn et al. 2011). They found that whereas *q*_L_‐based and *A*_n_‐based models had similar prediction accuracy for *g*_s_ measured at a leaf level, the fitted parameters of the *q*_L_‐based model were more consistent across varying measurement conditions. Even the similarity in prediction accuracy itself should be of significance: *q*_L_ and *g*_s_ are two entirely independent measurements, whereas *A*_n_ and *g*_s_ are not independent measurements at the leaf level, and a good correlation is inherent between the latter measured pair.

Our understanding of photosynthetic regulations summarized above points to the importance of feedback and feedforward mechanisms in ensuring the balance among photophysical, photochemical, and biochemical reactions. As part of the efforts to understand such mechanisms, we investigate how the processes that occur during the intermediate stage of photosynthesis—the photochemical reactions—are related to those in the upstream (the photophysical reactions) as well as those in the downstream (the biochemical reactions). More specifically, we would like to determine how the redox states of PSII, PQ, and Cyt and the thylakoid swelling/shrinking covary with NPQ, which regulates the photophysical reactions, and *g*_s_, which regulates the biochemical reactions, across species and climates. Answering this question is essential to predictively modeling photosynthesis as a complete system.

Major challenges in addressing the question above lie in a lack of means to simultaneously quantify the redox states of different ETC components and the ultrastructural dynamics of thylakoid in conjunction with measurements of NPQ and *g*_s_ in living plants under physiologically relevant conditions. Previous studies have used the redox state of PSII (e.g., *q*_L_), which can be monitored with pulse amplitude modulated (PAM) fluorometry, to approximate the redox states of PQ and Cyt (e.g., Kramer et al. 2004; Głowacka et al. 2018; Johnson and Berry 2021; Kromdijk et al. 2019). The accuracy of such approximations is questionable (Diner 1977). The redox state of a reactant depends on its abundance and the specific reactions it participates, so there are no obvious reasons for the redox states of PSII, PQ, and Cyt to be identical. Under steady state, how close the redox state of PSII can be used to approximate those of PQ and Cyt must be related to their stoichiometry as well as the environmental conditions that affect different electron flow pathways (e.g., linear vs. cyclic electron transport). The stoichiometry of protein complexes on the ETC varies with species and environmental conditions (Chow et al. 1990; Schöttler and Tóth 2014; McKenzie et al. 2020). The ETC has a two‐electron gate at PQ that needs to accept two electrons before becoming mobile and able to deliver electrons to Cyt, whereas the acceptor of PSII can only accept one electron at a time (Stirbet et al. 2020). PSII is involved in linear electron transport, whereas other components of the ETC (e.g., PQ, Cyt, plastocyanin, and PSI) are involved in both linear and cyclic electron transport whose relative proportion must be adjusted in response to environmental variations to keep the energy supply and demand in balance (Kramer and Evans 2011). Taken together, all these factors mean that the redox state equivalence of PQ and Cyt with PSII is unlikely to be true in general. High‐performance liquid chromatography (HPLC) can be applied on homogenized leaf extracts to monitor the redox state of the plastoquinone pool (Kruk and Karpinski 2006). However, HPLC cannot be applied to living plants in natural environments. Although electron microscopy has been used to demonstrate dark–light contrasts of thylakoid ultrastructure (e.g., Li et al. 2020), continuous monitoring of thylakoid ultrastructure of a chloroplast in vivo is currently technologically challenging. It may be possible to use neutron scattering techniques (Qian et al. 2020; Nagy and Garab 2020) or circular spectropolarimetric signals (Patty et al. 2019) to monitor the ultrastructural dynamics of thylakoid in vivo in response to systematic variations in environmental conditions. This approach remains to be tested. The presence of these challenges forces us to look for alternative approaches to studying regulatory coordination in photophysical, photochemical, and biochemical reactions.

With the recent development of mechanistic steady‐state photochemical model of redox reactions of photosynthetic electron transport (Gu et al. 2023), it is now possible, although direct confirmation and therefore caution in application are still needed, to infer the redox states of PQ and Cyt as well as the dynamics of thylakoid swelling/shrinking from PAM fluorometry measurements. These inferences can then be related to the corresponding measured values of stomatal conductance and NPQ to develop hypotheses for further testing. Gu et al. (2023) developed and tested two different photochemical redox models of photosynthetic linear electron transport. One is based on the dichotomous open‐closed (OC) representation of PSII reaction centers (the OC model), and the other is based on the redox states of the primary (Q_A_) and secondary (Q_B_) quinone acceptors of PSII (the Q_A_Q_B_ model). These two models are built upon earlier efforts in modeling linear electron transport to simulate the rapid or slow chlorophyll fluorescence induction curves (e.g., Ebenhöh et al. 2011; Laisk et al. 2006; Lazár 2013; Loriaux et al. 2013; Zhu et al. 2005, 2013). These earlier models use ordinary differential equations to describe the temporary variations of redox reagent pool sizes (states) and the fluxes between them. These equations cannot be solved analytically, even in steady state. They also contain numerous parameters, many of which cannot be estimated at the leaf scale in field conditions. In contrast, the OC and Q_A_Q_B_ models of Gu et al. (2023) can be solved analytically in steady state and their parameters can be estimated from PAM fluorometry measurements taken in the field. We note that there are simpler, less mechanistic models of light response of electron transport (e.g., Yang et al. 2025). These models are easier to use but mix photophysical and photochemical reactions, making them not particularly suitable for the study proposed here. The OC and Q_A_Q_B_ models have been developed specifically for photochemical reactions, and they complement the photophysical model of Gu et al. (2019). The OC and Q_A_Q_B_ models perform almost equally well in fitting leaf‐level measurements. However, the Q_A_Q_B_ model is more complex with more parameters and can potentially overfit the measurements. The OC has been applied to determine how the ETC can be bioengineered to improve the electron transport capacity while simultaneously minimizing the risk of over‐reduction and photodamage (Gu 2023). In this study, we apply the OC model. By joining OC model‐inferred PQ and Cyt redox states and thylakoid ultrastructural dynamics with simultaneous measurements of NPQ, *q*_L_, and *g*_s_, we are able to explore, for the first time, how regulations of photophysical, photochemical, and biochemical reactions are coordinated to ensure the safe operation of the photosynthetic apparatus in dynamic environments.

## 2 Materials and Methods

We took advantage of a large dataset of joint measurements of PAM fluorometry and gas exchange of light, CO_2_, O_2_, and temperature responses collected from climates ranging from boreal to the tropics. These data were used to determine the variables of *NPQ*, *q*_*L*_, the linear electron transport rate (*J*_PSII_), and *g*_s_. We then applied the OC model to the data of *q*_L_ and *J*_PSII_ to infer the redox states of PQ and Cyt and a light‐induced thylakoid swelling/shrinking function. The redox state of PQ is quantified by the oxidized fraction of the mobile PQ pool, denoted by *h*_PQ_. The redox state of Cyt is quantified by the fraction of the Cyt complexes available for linear electron transport, denoted by *h*_cyt_. The thylakoid swelling/shrinking function (*f*_s_) is a unitless scalar that can theoretically vary between 0 and 1. The unlikely theoretical value of *f*_s_ = 0 indicates that the thylakoid is shrunk to such a small volume and the macromolecular blocking is so complete that no electron carriers can move around. The theoretical value of *f*_s_ = 1 indicates that the thylakoid is fully swollen and that integral protein complexes presents no structural blocking to the diffusion of electron carriers.

Ideally, directly measured *h*_PQ_, *h*_cyt_, and *f*_s_, rather than their model‐inferred values, should be used in the study. However, currently, no experimental techniques are available to systematically monitor the responses of *h*_PQ_, *h*_cyt_, and *f*_s_ to variations in environmental conditions. Rather than waiting for new measurement techniques to emerge and doing nothing, the inference by mechanistic models offers the next best approach. We will carefully examine the model‐inferred *h*_PQ_, *h*_cyt_, and *f*_s_ against all available empirical evidence we could find to determine their reasonableness. Only after the reasonableness of the inferred values is established, we proceed with their analyses. We hope our study will promote development of experimental techniques to monitor the dynamics of *h*_PQ_, *h*_cyt_, and *f*_s_ in natural environments.

### 2.1 Datasets and Measurement Protocols Used in the Study

The dataset and measurement protocols used in this study were fully documented in Gu et al. (2023) and Han et al. (2022) and are publicly available from www.leafweb.org. Here only a brief summary is provided. Simultaneous measurements of PAM fluorometry and gas exchange of light, CO_2_, O_2_, and temperature responses were made on more than two dozens of C_3_ and C_4_ species in Canada, China, the Netherlands, and the United States. These species are distributed in climates ranging from the boreal to the tropics. They include lianas, shrubs, boreal deciduous and evergreen needle‐leaf trees, temperate deciduous trees, tropical deciduous and evergreen trees, C_3_ and C_4_ grasses, and crops. At the time of measurements, no species were suffering from obvious stresses (e.g., drought and extreme temperatures). As the data were collected by different laboratories in various countries, different instruments were used. Most of the species were measured with either Li 6400 or 6800, but some were measured with GFS‐3000. Which species was measured by which instrument was recorded in the public data release document (Han et al. 2022). These measurements followed standard protocols for PAM fluorometry (Baker 2008) and gas exchange (Long and Bernacchi 2003) with each leaf dark‐adapted for at least 30 min before any measurement started. All species were measured with light responses with the ambient CO_2_ concentration controlled at a constant level (e.g., 400 ppm). A majority of species were also measured with CO_2_ responses. Measurement temperatures were either controlled at a constant value (e.g., 21°C or 25°C) or varied between two extremes (e.g., 9°C–40°C). Most measurements were made at ambient O_2_ concentration although for some species, multiple oxygen levels (from 2% to 50%) were also used. All PAM fluorometry measurements started with fully dark‐adapted leaves to determine the minimum (F_0_) and maximum (F_M_) fluorescence yield. The photochemical quantum yield of PSII (*Φ*_PSII_) was calculated as $1-\frac{F_{s}}{F_{M}^{\prime }}$ as in standard fluorometry (Genty et al. 1989) where *F*_s_ is the steady state fluorescence yield in the daytime or with the actinic light on and $F_{M}^{\prime }$ is the corresponding maximum fluorescence yield obtained with a saturation pulse. *J*_PSII_ is calculated with $J_{\text{PSII}}=\Phi_{\text{PSII}}\times \text{αβPAR}$. α = 0.85 is the leaf absorptance in PAR, and β = 0.5 is the fraction of absorbed PAR allocated to PSII. The fraction of open PSII reaction centers was calculated with either the lake or puddle model connectivity of photosynthetic units (Kramer et al. 2004). $F_{0}^{\prime }$, which is the minimum fluorescence yield with all PSII reaction centers open but NPQ unrelaxed and needed for the determination of the fraction of open PSII reaction centers, is calculated with the Oxborough–Baker approach (Oxborough and Baker 1997). The NPQ variable is calculated as $\mathrm{NPQ}=\frac{F_{M}}{F_{M}^{\prime }}-1$.

### 2.2 Quantifying the Redox States of PSII, PQ, and Cyt and the Thylakoid Swelling and Shrinking

The OC model used in the present study was derived and described fully in Gu et al. (2023), which validated the model with the leave‐one‐out cross‐validation approach against measurements from about two dozens of species. Gu et al. (2023) provided a simple but convenient Excel Spreadsheet tool implemented with the OC model. That tool, which employs the evolutionary optimization approach available in Excel, could be used by independent investigators to verify the results of this paper or conduct their own analyses (but see the next section for caution). Gu et al. (2023) performed sensitivity tests with the OC model to assess the importance of different process representations. Its supplementary materials included statistics about parameters estimated and their ranges. The analyses and tests conducted in Gu et al. (2023) showed that the OC model contains no redundant parameters and has excellent parameter estimability with typical PAM fluorimetry measurements following standard protocols. Readers are referred to Gu et al. (2023) for model details. Equations relevant to the present study are presented below. According to the OC model, the photochemical relationship between the linear electron transport rate (*J*_PSII_) and the fraction of open PSII reaction centers (*q*) under either the lake or puddle model connectivity of photosynthetic units is governed by the following equation:

(1) $$J_{\text{PSII}}=\frac{2Uf_{T}f_{s}f_{q}\left(q_{r}-q\right)q}{\left(R_{1}+2R_{2}f_{s}f_{q}-1\right)q+q_{r}}.$$

Here *U* = $uN_{{\mathrm{cyt}}_{T}}N_{{\mathrm{PQ}}_{T}}$ with *u* the second‐order rate constant for the oxidation of plastoquinol (PQH_2_) by the Rieske FeS protein of Cyt and the accompanying transport of proton from the stroma to the lumen, $N_{{\mathrm{cyt}}_{T}}$ the foliar concentration of Cyt for linear electron transport, and $N_{{\mathrm{PQ}}_{T}}$ the total foliar concentration of mobile plastoquinone (oxidized and reduced) for linear electron transport. *R*_1_ = *r*_r_/*r*_d_ with *r*_d_ and *r*_r_ being the second‐order rate constant for the electron transfer from the reduced acceptor to plastoquinone to form PQH_2_ and for the reverse reaction, respectively. $R_{2}=\frac{u}{r_{d}}\times \frac{N_{{\mathrm{cyt}}_{T}}}{N_{\text{PSII}}}$ with *N*_PSII_ the foliar concentration of PSII reaction centers whose functional reversible fraction is denoted by *q*_r_. *f*_*T*_ is the standardized temperature response function for the rate constants of redox reactions. It is derived from the Marcus theory of electron transfer in proteins and given by $\sqrt{\frac{T_{0}}{T}}e^{E_{T}\left(\frac{1}{T_{0}}-\frac{1}{T}\right)}$. *E*_*T*_ is the temperature (*T*) sensitivity parameter related to the Gibbs free energy of activation, and *T*_0_ is the reference temperature. *f*_q_ quantifies the redox poise balance between PSII and Cyt and is given by

(2) $$f_{q}=\frac{1+a_{q}}{1+a_{q}\times q}.$$

*a*_*q*_ is a PSII–Cyt stoichiometry parameter (see Gu et al. 2023 and Gu 2023 for discussion). The redox state of Cyt is indicated by its fraction available for oxidizing PQH_2_, denoted by *h*_cyt_:

(3) $$h_{\mathrm{cyt}}=f_{q}\times q.$$

The light‐induced thylakoid swelling/shrinking function, denoted by *f*_s_, is given by

(4) $$f_{s}=\frac{V}{V_{\max}}=\frac{1}{1+c_{s}e^{-b_{s}\times \text{αPPFD}}}.$$

Here, *V* is the total volume of the thylakoid at a given level of absorbed photosynthetic photon flux density (*αPPFD*, *α* being leaf absorptance), and *V*_*max*_ is the maximum thylakoid volume when it is fully swollen. *b*_*s*_ and *c*_*s*_ are two empirical coefficients with *b*_*s*_ controlling how fast the thylakoid expands and *c*_*s*_ setting the maximum impact of macromolecular crowding on the diffusion of PQ and PC. As shown in Gu et al. (2023), the redox state of the PQ/PQH_2_ pool is described by

(5) $$h_{\mathrm{PQ}}=\frac{R_{1}+2R_{2}f_{s}f_{q}}{\left(R_{1}+2R_{2}f_{s}f_{q}-1\right)q+q_{r}}q.$$

All parameters of the redox model can be estimated by fitting Equation (1) to PAM fluorometry measurements, for example, using the Excel Spreadsheet‐based tool offered in Gu et al. (2023). Equation (1) is valid regardless of the connectivity of photosynthetic units because it governs the post‐charge separation electron transport along the ETC, whereas photosynthetic unit connectivity concerns whether and how excitons can be shared among different photosynthetic units, which occurs prior to charge separation. For a discussion on different models of photosynthetic unit connectivity, see Kramer et al. (2004) and Blankenship (2014).

Within the redox model of photosynthetic electron transport, $f_{s}$ represents the thylakoid ultrastructural impact on the diffusion of PQ and PC and therefore the electron transport. This is achieved by using *f*_*s*_ to modify *h*_cyt_. In the dark, the thylakoid shrinks to a minimum volume, corresponding to a minimum *f*_s_. Under this condition, the macromolecular restriction on the diffusion of PQ and PC is maximal, and the thylakoid ultrastructure has the maximal impact on *h*_cyt_. In the light as the thylakoid swells, likely due to osmotic water influx into lumen, *f*_s_ increases, and the thylakoid ultrastructure relaxes its restriction on the diffusion of PQ and PC, allowing these electron carriers to move more freely. Under saturating light conditions, the thylakoid swells to the maximum, *f*_s_ approaches 1, and the restriction of the thylakoid ultrastructure on the diffusion of PQ and PC reaches minimum. Short of an ideal test of the bellows theory against continuous electron microscopy measurements, the predictions made by the bellows theory can be tested by comparing *f*_s_ inferred from PAM fluorometry measurements against the corresponding observations of NPQ and stomatal conductance for water vapor.

### 2.3 Optimization Method for Inferring Photochemical Parameters and *f*_s_

We used a hybrid global optimization algorithm to fit Equation (1) to PAM fluorometry measurements to infer photochemical model parameters and *f*_s_. This global optimization algorithm applied evolutionary method, gradient descent, and compass search in sequence with repeated random guess reinitialization to avoid the search being trapped in local minima (Gu et al. 2023). The fitting was based on either the lake or puddle model for the fraction of open PSII reactions, denoted by *q*_L_ or *q*_P_, respectively. As in Gu et al. (2023) who found that the performance of Equation (1) was equally well between these two models of photosynthetic unit connectivity, we found that the testing results for the predictions of the bellows theory do not depend on photosynthetic unit connectivity. Here, we only report results based on the lake model. The optimization approach used in this study is somewhat more sophisticated than the evolutionary optimization approach available in Excel. Users of the Excel Spreadsheet tool in Gu et al. (2023) should check their optimization to make sure that global minima have been reached, for example, by choosing different initial guesses and by running the tool multiple times in sequence.

## 3 Results

### 3.1 Variations of the Inferred Redox States of ETC and Thylakoid Ultrastructural Dynamics

Light intensity exerts a feedforward control of the redox states of the ETC from the photophysical reactions to the photochemical reactions, whereas the intercellular CO_2_ concentration (*C*_i_) applies a feedback control from the biochemical reactions to the photochemical reactions; both can either independently or interactively affect the redox states of the ETC. For a given ambient CO_2_ concentration, *q*_L_, *h*_PQ_, and *h*_cyt_ all decrease with increased photosynthetically active light intensity for all species included in this study (Figure S1). The decreases are steeper at low than high light intensities such that at the end of high light intensity, the change is almost linear. For a given light intensity, *q*_L_, *h*_PQ_, and *h*_cyt_ all increase with increased *C*_i_ (Figure S2), but the increase is only up to a value of *C*_*i*_ beyond which the curve flattens or even starts to decrease slightly (e.g., Figure S2E). As a feedforward control, variations in light intensity can manipulate the redox states of the ETC within the full range expected (i.e., fully reduced to fully oxidized, 0 < *q*_L_, *h*_PQ_, and *h*_cyt_ < 1). In contrast, as a feedback control, the degree to which the redox states of the ETC can be manipulated by varying *C*_i_ depends on the light intensity with wider ranges achievable at low than high light intensities (e.g., comparing the plot A with the remaining plots in Figure S2).

**FIGURE 1:**
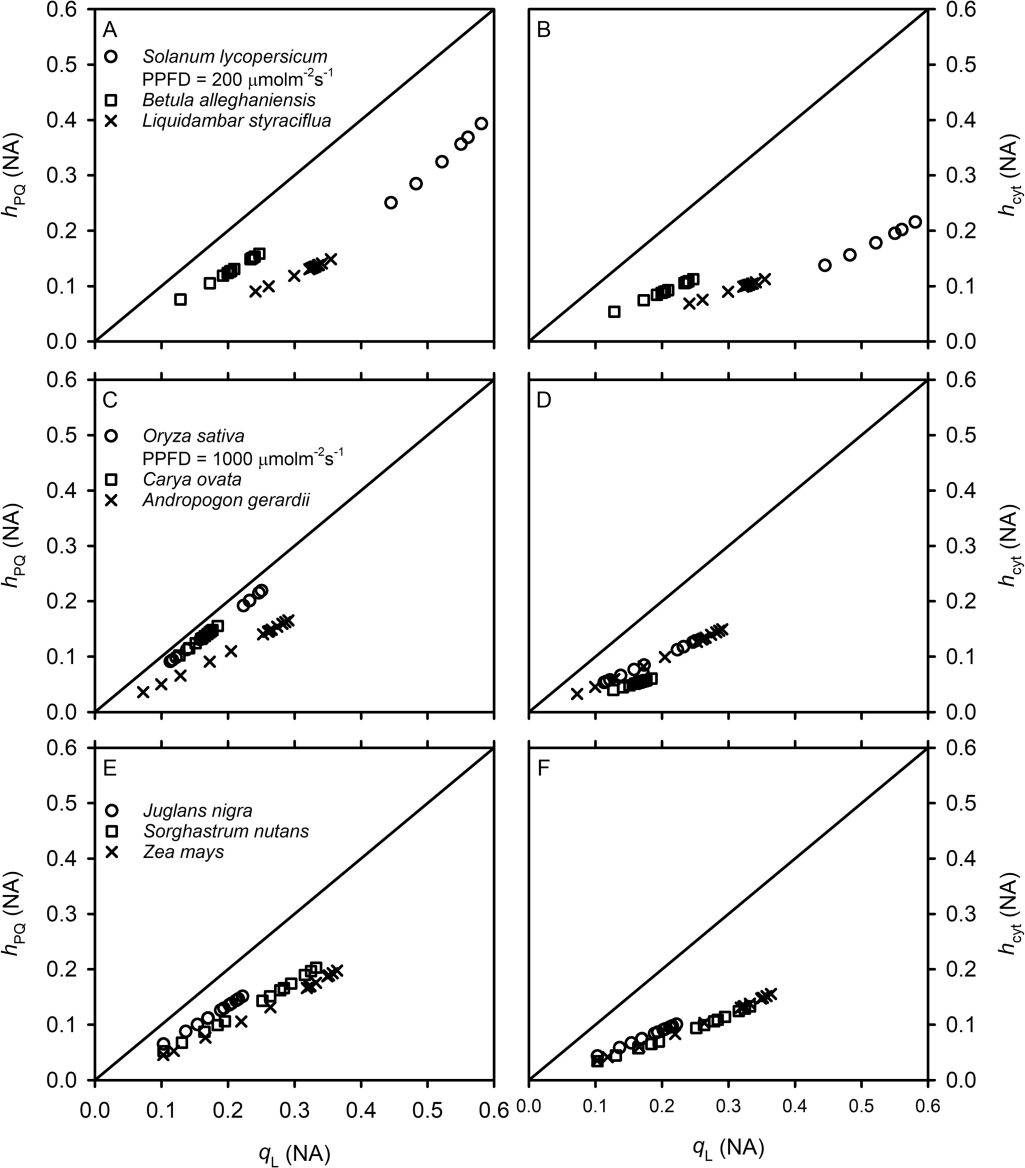
The CO_2_‐induced redox state relationships between PSII, plastoquinone, and cytochrome b_6_f complex. The oxidized fraction of mobile plastoquinone pool (*h*_PQ_) and the fraction of cytochrome b_6_f complex available for linear electron transport (*h*_cyt_) are plotted against the fraction of open photosystem II reaction centers under the assumption of lake model (*q*_L_) for CO_2_ response curves of different species under constant photosynthetic photon flux densities (PPFD). For all species, the PPFD is 2000 μmol m^−2^ s^−1^ unless otherwise specified. The 1:1 line is also shown.

The redox states of PSII, PQ, and Cyt are synchronized only in terms of the general direction they vary, that is, *q*_L_, *h*_PQ_, and *h*_cyt_ decrease or increase together as environmental conditions vary but their trajectories are not identical, as demonstrated in Figures S1 and S2. Overall, Cyt tends to be more reduced than PQ, whereas PQ tends to be more reduced than PSII (i.e., *h*_cyt_<*h*_PQ_ < *q*_L_). This is particularly true when the ETC is strongly reduced (i.e., small values of *q*_L_, *h*_PQ_, and *h*_cyt_ in Figure S3 and in Figure 1). When the ETC is strongly oxidized, *h*_PQ_ and *h*_cyt_ can be higher than *q*_L_ (e.g., Figure S3A,C,E).

**FIGURE 2:**
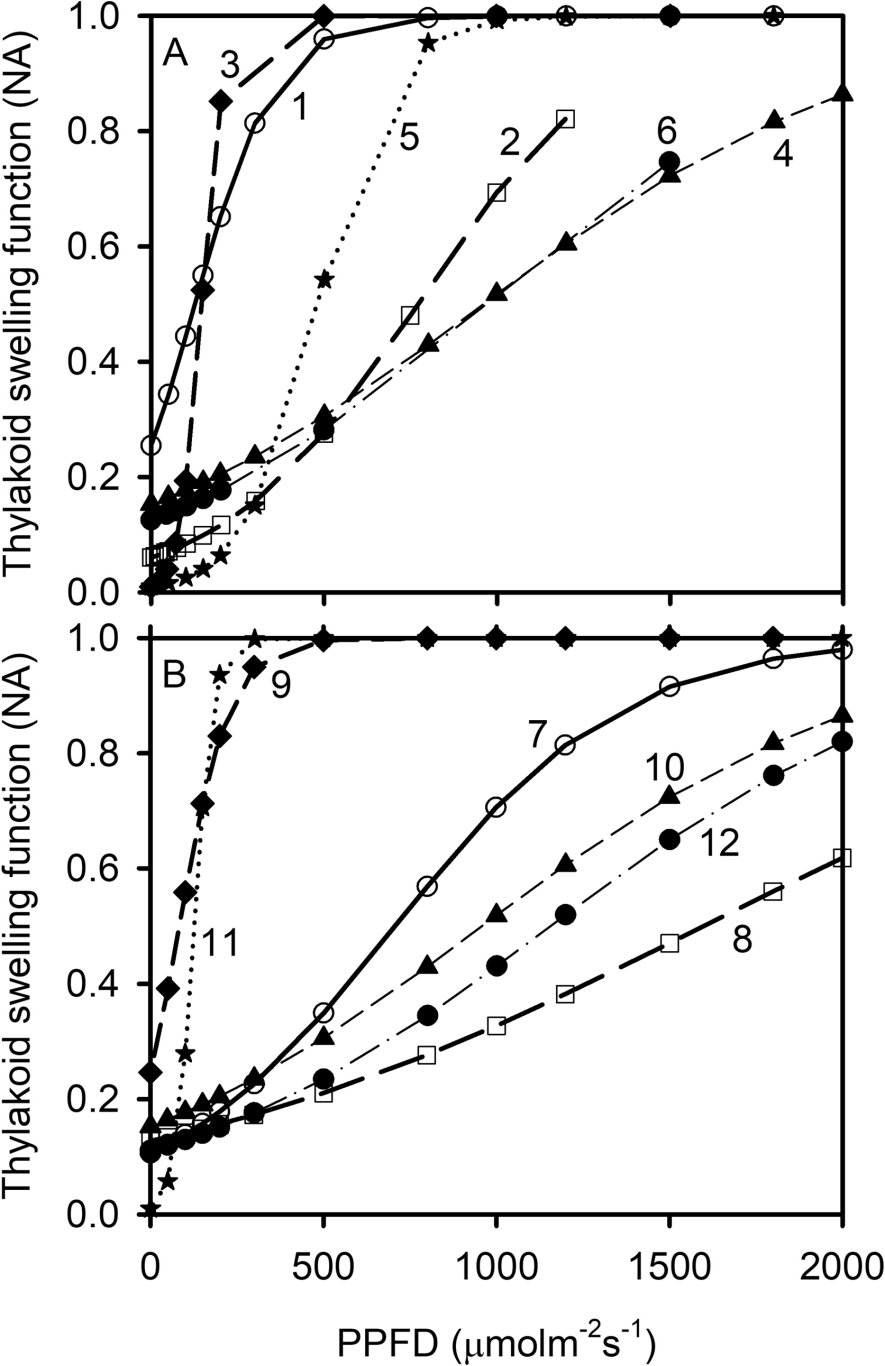
The thylakoid swelling as a function of photosynthetic photon flux density. Each curve is marked with a number in A and B and represents a species/cultivar: 1, *Bauhinia glauca*; 2, *Solanum lycopersicum* , tomato Basket Vee; 3 *S. lycopersicum* , tomato Growdena; 4, *Zea mays* ; 5, *Bauhinia purpurea* ; 6, *Oryza sativa* , rice IR64; 7, *Cornus racemosa* “Ottzam”; 8, *Betula alleghaniensis* ; 9, *Magnolia henryi*; 10, *Juglans nigra* ; 11, *Dichanthelium clandestinum* ; 12, *Sorghastrum nutans* .

The mathematical formulation of the *f*_*s*_ function and its optimization against PAM fluorometry measurements do not prescribe a general trend between *f*_*s*_ and light intensity. In fact, using appropriate values of *b*_*s*_ and *c*_*s*_, *f*_*s*_ can increase, decrease, or show no change at all with light intensity. Yet, the obtained values of *b*_*s*_ and *c*_*s*_ that produce the best fit are such that the optimized *f*_*s*_ function increases with light intensity for all species (Figure 2; also, table S3 in Gu et al. 2023; table 2 in Gu 2023). For some species, *f*_s_ increases rapidly and reaches a plateau, whereas for others, *f*_s_ increases almost linearly with light intensity.

### 3.2 Empirical Support for Our Model Inferences

Currently, no methods are available allowing nondestructive continuous monitoring of the redox states of PQ and Cyt as well as the thylakoid ultrastructural dynamics on leaves of living plants under field conditions. However, different laboratory techniques have been developed to process leaf samples and monitor the state conditions of the ETC that are also inferred by the photochemical model used in our study. Here, we compare the general patterns reported in previous experimental studies with those inferred by this study to evaluate the validity of the model. Cleland (1998) used a voltammetric technique with a “Q” electrode to monitor the redox state of PQ in isolated thylakoids and found that the voltage change of the electrode (the Q signal), which was indicative of the reduced PQ pool size, nonlinearly increased with light intensity and leveled off at high light. Kruk and Karpinski (2006) applied the HPLC method to homogenized leaf extracts and found that the plastoquinone pool was more reduced in the light than in the dark. These two earlier studies were confirmed in a more recent experiment, also applying the HPLC method by Mattila et al. (2020), which showed that the redox state of PQ could effectively serve as a proxy for light intensity. A more direct confirmation of our model inference can be found in Yoshida et al. (2010). These researchers applied the HPLC method to plastoquinone extracted from *Arabidopsis thaliana* and found that its reduction level increased under high‐light treatments versus low‐light treatments. The remarkable consistency of findings of our study with those of Cleland (1998), Kruk and Karpinski (2006), Yoshida et al. (2010), and Mattila et al. (2020) provides strong support for using the OC model and PAM fluorometry measurements to infer the redox states of the ETC components.

We are not aware of any previously measured responses of the redox states of the ETC to variations in CO_2_ concentration. However, our model‐inferred responses (Figure S2) are consistent with the well‐tested FvCB biochemical model of photosynthesis. The initial rapid increases in *q*_L_, *h*_PQ_, and *h*_cyt_ with the increased *C*_i_ at a constant light level correspond to increased Rubisco carboxylation and therefore heightened demand for electron transport products, which leads to the oxidation of the ETC. As *C*_i_ continues to increase, however, carboxylation is transitioned to limitation by RuBP regeneration, halting the continual oxidation of the ETC. Further increases in *C*_i_ may result in carboxylation being limited by triose phosphate utilization (Sharkey 1985), causing slight re‐reduction of the ETC in some species (e.g., Figure S2E).

The model‐inferred redox relationships between different components of the ETC are also consistent with previous experimental findings. Diner (1977) used fluorescence induction and isolated thylakoids to test the previously held assumption that the redox states were proportional between Q_A_ (the primary electron acceptor of PSII) and PQ. Diner found that this assumption was incorrect; in fact, PQ was reduced faster than Q_A_. Similar results were obtained in Cleland (1998). Our model‐inferred relationships between *h*_PQ_ and *q*_L_ (Figure S3A,C,E) agree with Diner and Cleand's experimental findings. Yoshida et al. (2010) again provided more convincing evidence supporting the inferred redox relationships. They found that the reduction level of plastoquinone was higher than and increased with 1–*q*_P_ (figure 3B in Yoshida et al.). By comparing figure 3B in Yoshida et al. with Figures 1 and S3 in the present study, we see that our model inference also agrees with Yoshida et al.'s HPLC data. Yoshida et al. used the puddle model (*q*_P_), whereas our study applies the lake model (*q*_L_). As we stated earlier, these two models do not affect our results qualitatively. With respect to the redox state of Cyt, our model infers that under a given set of environmental conditions, *h*_cyt_ is generally less than either *q*_L_ or *h*_PQ_ (Figures S1–S3). This is consistent with the experimental suggestion that the oxidation of PQH_2_ by Cyt is the rate‐limiting step of electron transport (Laisk et al. 2005; Rochaix 2011).

The model‐inferred patterns of the thylakoid swelling/shrinking function *f*_s_ are qualitatively consistent with the electron microscopy observations of light‐induced change in thylakoid volume (Packer et al. 1965; Murakami and Nobel 1967; Krause 1973; Kirchhoff et al. 2011; Kirchhoff 2014). In some studies, an expansion of lumen width exceeding the size of plastocyanin has been observed (Li et al. 2020). Although the mechanisms of thylakoid ultrastructural dynamics are in much‐needed studies, lumen acidification and the associated ion movements resulting from electron transport can lead to disequilibrium in water potential between the lumen and stroma, which in turn drives osmotic water fluxes across the thylakoid membrane, providing the force for thylakoid swelling/shrinking (Beebo et al. 2013; Li et al. 2020). Model simulations conducted by Kirchhoff et al. (2011) showed that the observed magnitude of light‐induced expansion of thylakoid volume can greatly reduce structural restrictions on the diffusion of electron carriers. These observational and structural modeling results are fully consistent with the roles that the inferred *f*_s_ function plays in the photochemical model used in our study.

Given the general agreement of the model‐inferred redox states of ETC and thylakoid ultrastructural dynamics with previous experimental and modeling studies, we now have confidence to couple measurements and modeling to investigate how regulatory mechanisms of photophysical, photochemical, and biochemical reactions are coordinated in the photosynthesis of land plants.

### 3.3 Variations of Redox States of ETC and Thylakoid Swelling/Shrinking With Non‐Photochemical Quenching

We examined how the parameter *NPQ* is related to the variations of redox states of ETC and thylakoid swelling/shrinking. *NPQ*, rather than the quantum yield of NPQ, denoted by *Y* (NPQ) (Klughammer and Schreiber 2008; Lazár 2015), was used in this analysis because the former is entirely determined by the processes of non‐photochemical quenching, whereas the latter is additionally affected by the processes of photochemical quenching. This can be seen by expressing the parameter *NPQ* and *Y* (NPQ) in rate constants. *NPQ* = *k*_n_/(*k*_f_ + *k*_d_) and *Y* (NPQ) = *k*_n_/(*k*_f_ + *k*_d_ + *k*_n_ + *k*_p_), here *k*_f_, *k*_d_, *k*_n_, and *k*_p_ are the rate constants for fluorescence, constitutive heat dissipation, non‐photochemical quenching, and photochemistry, respectively. *k*_p_ appears in the expression for *Y* (NPQ) but not in that for *NPQ*. By using *NPQ*, we avoid ambiguity between photochemical and non‐photochemical processes.

**FIGURE 3:**
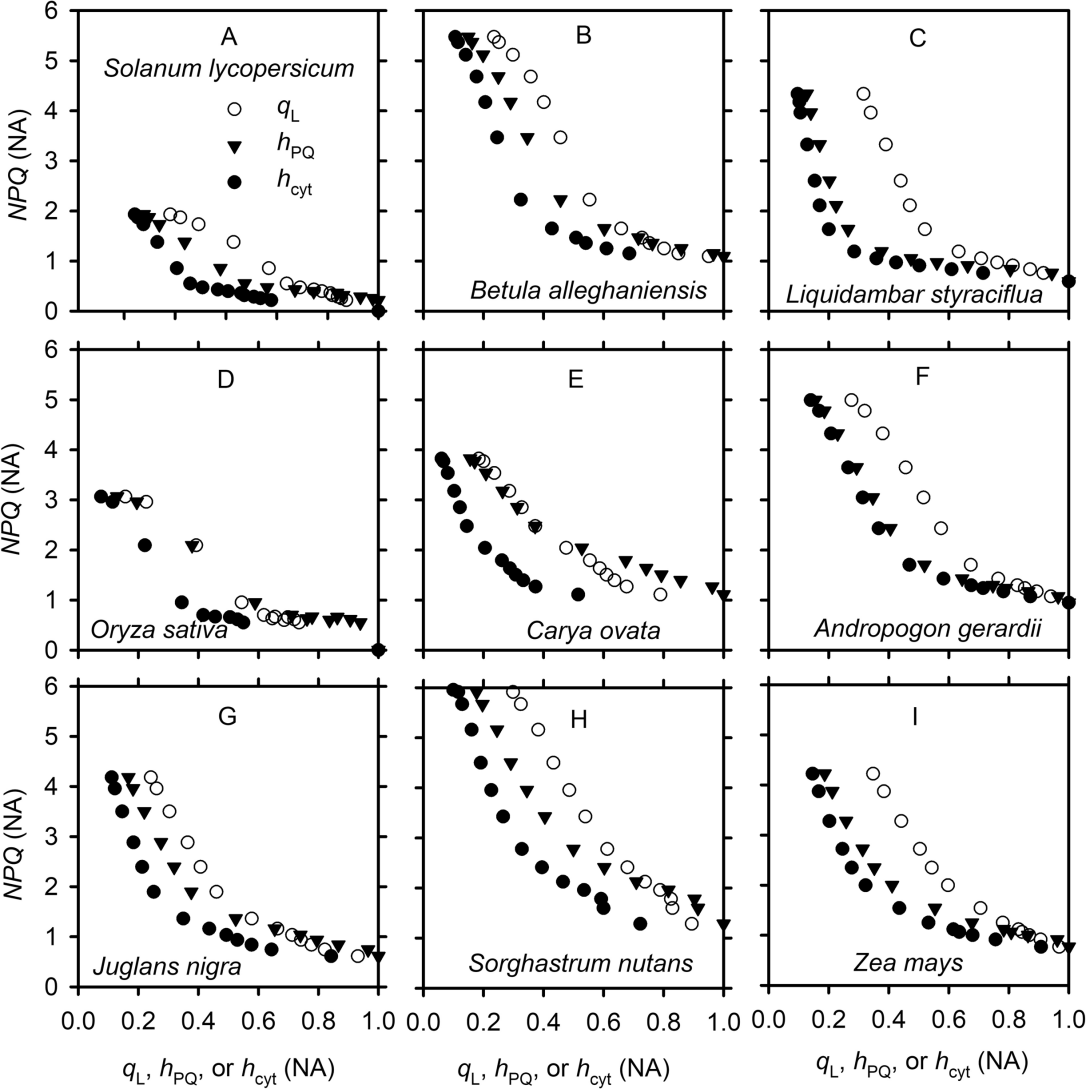
The light‐induced variations of *NPQ* with the redox states of PSII, plastoquinone, and cytochrome b_6_f complex. The variations in *NPQ*, the fraction of open photosystem II reaction centers under the assumption of lake model (*q*_L_, cycle), the oxidized fraction of mobile plastoquinone pool (*h*_PQ_, triangle), and the fraction of cytochrome b_6_f complex available for linear electron transport (*h*_cyt_, solid dot) are from the light response curves of different species under an ambient CO_2_ partial pressure of ~40 Pa.

**FIGURE 4:**
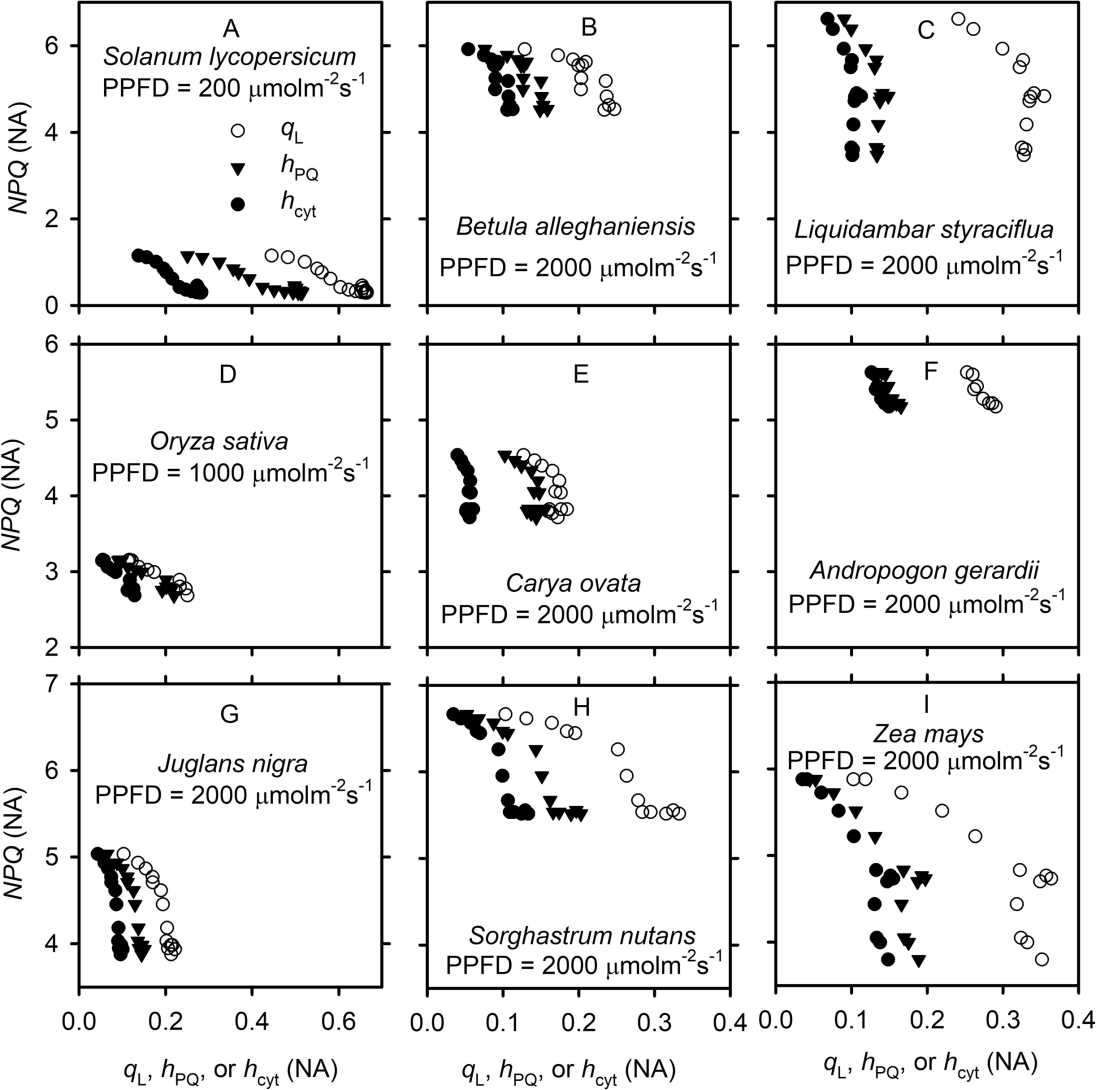
The CO_2_‐induced variations of *NPQ* with the redox states of PSII, plastoquinone, and cytochrome b_6_f complex. The variations in *NPQ*, the fraction of open photosystem II reaction centers under the assumption of lake model (*q*_L_, cycle), the oxidized fraction of mobile plastoquinone pool (*h*_PQ_, triangle), and the fraction of cytochrome b_6_f complex available for linear electron transport (*h*_cyt_, solid dot) are from the CO_2_ response curves of different species under different levels of photosynthetic photon flux density (PPFD).

*NPQ* decreases with increases in *q*_L_, *h*_PQ_, and *h*_cyt_, regardless of whether the dynamics were induced by variations in light intensity (Figure 3) or *C*_i_ (Figure 4). However, the patterns in their relationships differ sharply between those induced by variations in light intensity and those induced by *C*_i_. For the relationships induced by varying light intensities (Figure 3), *NPQ* initially decreases steeply as *q*_L_, *h*_PQ_, and *h*_cyt_ increase. This section of decrease corresponds to a reduced ETC. As the ETC transitions from being reduced to being oxidized, that is, as *q*_L_, *h*_PQ_, and *h*_cyt_ continue to increase, the decrease in *NPQ* becomes much gentler. For the relationships induced by varying *C*_i_ (Figure 4), the degree of variation in *NPQ* as well as in *q*_L_, *h*_PQ_, and *h*_cyt_ becomes much narrower, but the precise patterns depend on the light intensity. At low light intensity (e.g., Figure 4A), the variation of *NPQ* with *q*_L_, *h*_PQ_, and *h*_cyt_, induced by varying *C*_i_, has a reversed and slanted s shape. As *q*_L_, *h*_PQ_, and *h*_cyt_ increase (induced by increase in *C*_i_), the decrease of *NPQ* is initially gentle but becomes steep and then turns gentle again. In contrast, at high light intensities (e.g., Figure 4G), the decrease of *NPQ* is rather steep as the ETC becomes less reduced.

**FIGURE 5:**
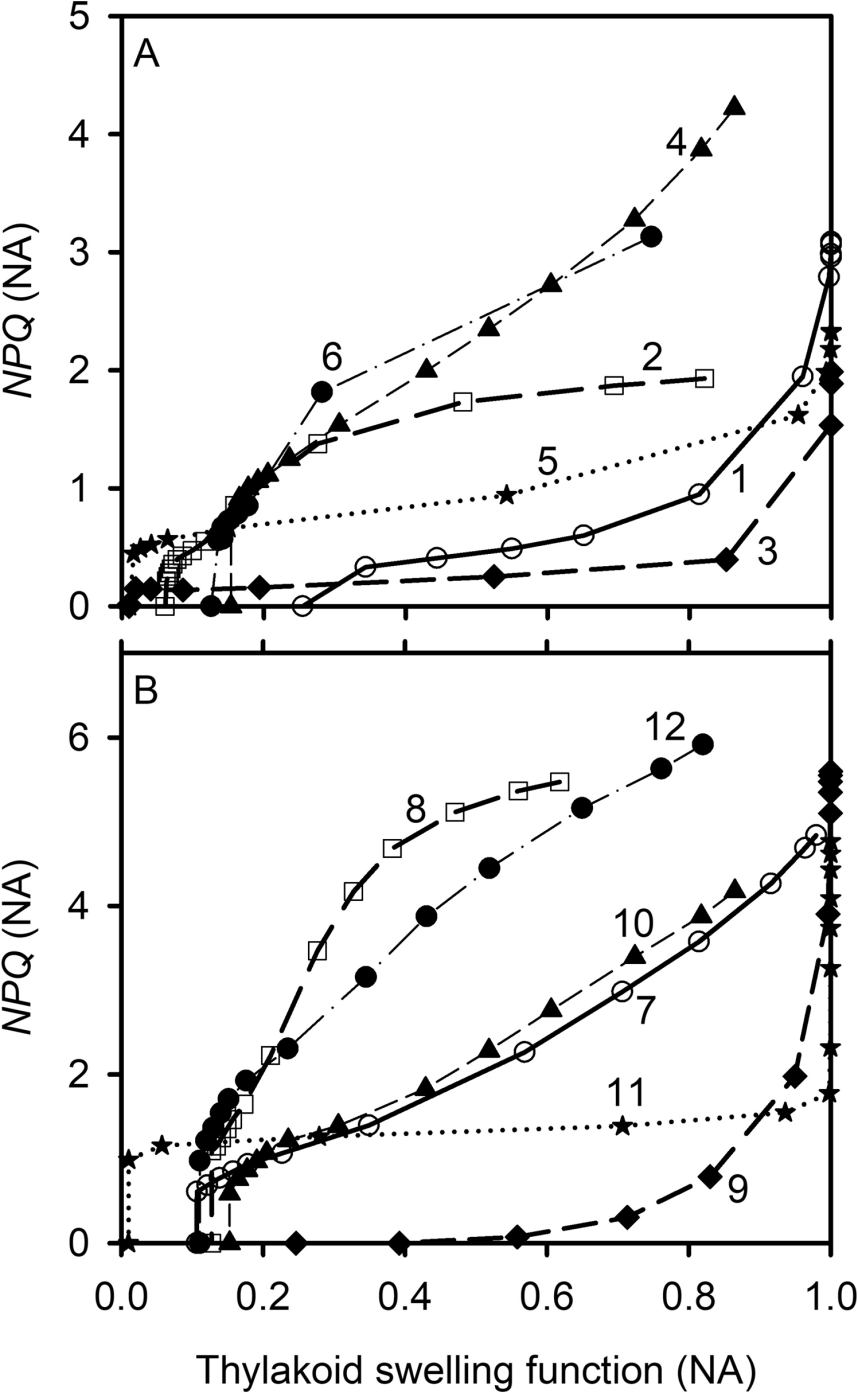
The relationship between *NPQ* and the thylakoid swelling function. Each curve is marked with a number in A and B and represents the same species/cultivar as shown in Figure 2.

**FIGURE 6:**
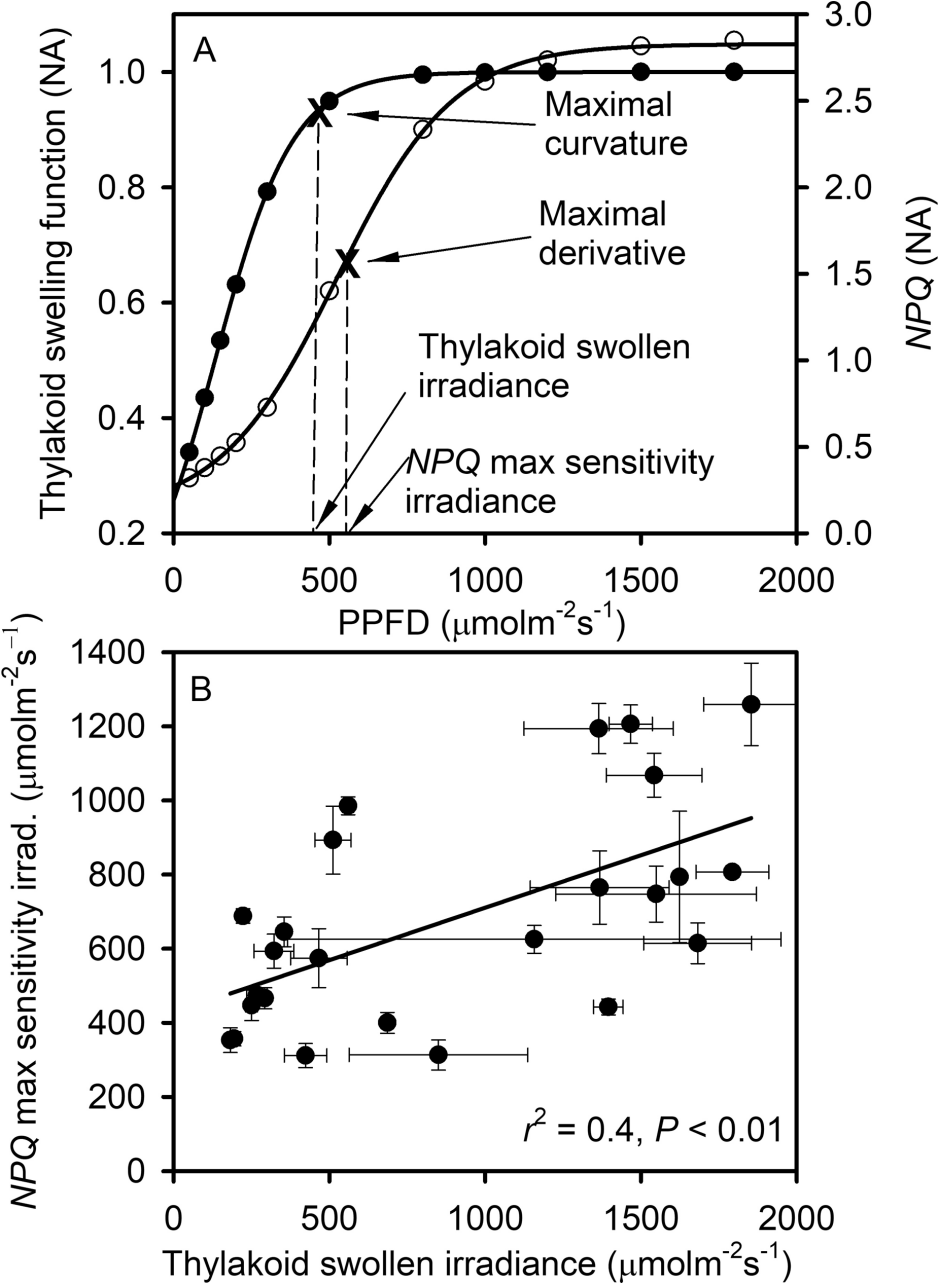
Relationships between the key characteristics of *NPQ* development and thylakoid swelling. This figure shows how the *NPQ* maximal sensitivity irradiance varies with the thylakoid swollen irradiance across species. As illustrated in A, the *NPQ* maximal sensitivity irradiance is the light intensity at which the *NPQ* light response (A, right axis) has the maximal derivative which is calculated from a sigmoid fitting to the measurements. The thylakoid swollen irradiance is the light intensity at which the thylakoid swelling function (A, left axis) has the maximal curvature before leveling off. In B, the vertical and horizontal bars represent one standard error averaged across the replicas of the same species. To avoid unreliable extrapolation, Plot B includes only response curves for which the maximal curvature of the thylakoid swelling function falls within the range of light intensity used in the measurements.

It is not straightforward to predict the relationship between *NPQ* and thylakoid dynamics because of the multiplicity and complexity of processes involved in the control of *NPQ* (Goss and Lepetit 2015) for which thylakoid swelling/shrinking is only one possible influencing factor (Gu et al. 2022). Nevertheless, we infer that as the PPFD increases to bring about steady‐state photosynthesis, thylakoids swell, while NPQ increases, resulting in a pattern of increased *NPQ* with *f*_s_ (Figure 5). The *NPQ* of most species initially increases rapidly as the thylakoid just begins to swell. But as the swelling continues, the increase in *NPQ* slows until the thylakoid approaches maximum expansion at which point *NPQ* increases rapidly again. To further investigate the relationship between *NPQ* development and thylakoid swelling, we calculated the *NPQ* maximal sensitivity irradiance, which is the irradiance at which *NPQ* has the maximal increase with light intensity. This calculation was done by first fitting a sigmoid function between *NPQ* and light intensity and then finding the maximal derivative of the fitted function. As shown in Figure 6A, the sigmoid function tightly fits the relationship between *NPQ* and light intensity. We also calculated the thylakoid swollen irradiance, which is defined as the irradiance at which the maximal curvature of *f*_s_ occurs, indicating that the thylakoid is approaching full expansion (Figure 6A). We found that, across species, the *NPQ* maximal sensitivity irradiance is statistically significantly and positively correlated with the thylakoid swollen irradiance (Figure 6B). This correlation suggests that the speed of *NPQ* development is affected by thylakoid swelling during the dark‐to‐light transition.

### 3.4 Variations of Redox States of ETC and Thylakoid Swelling/Shrinking With Stomatal Conductance

**FIGURE 7:**
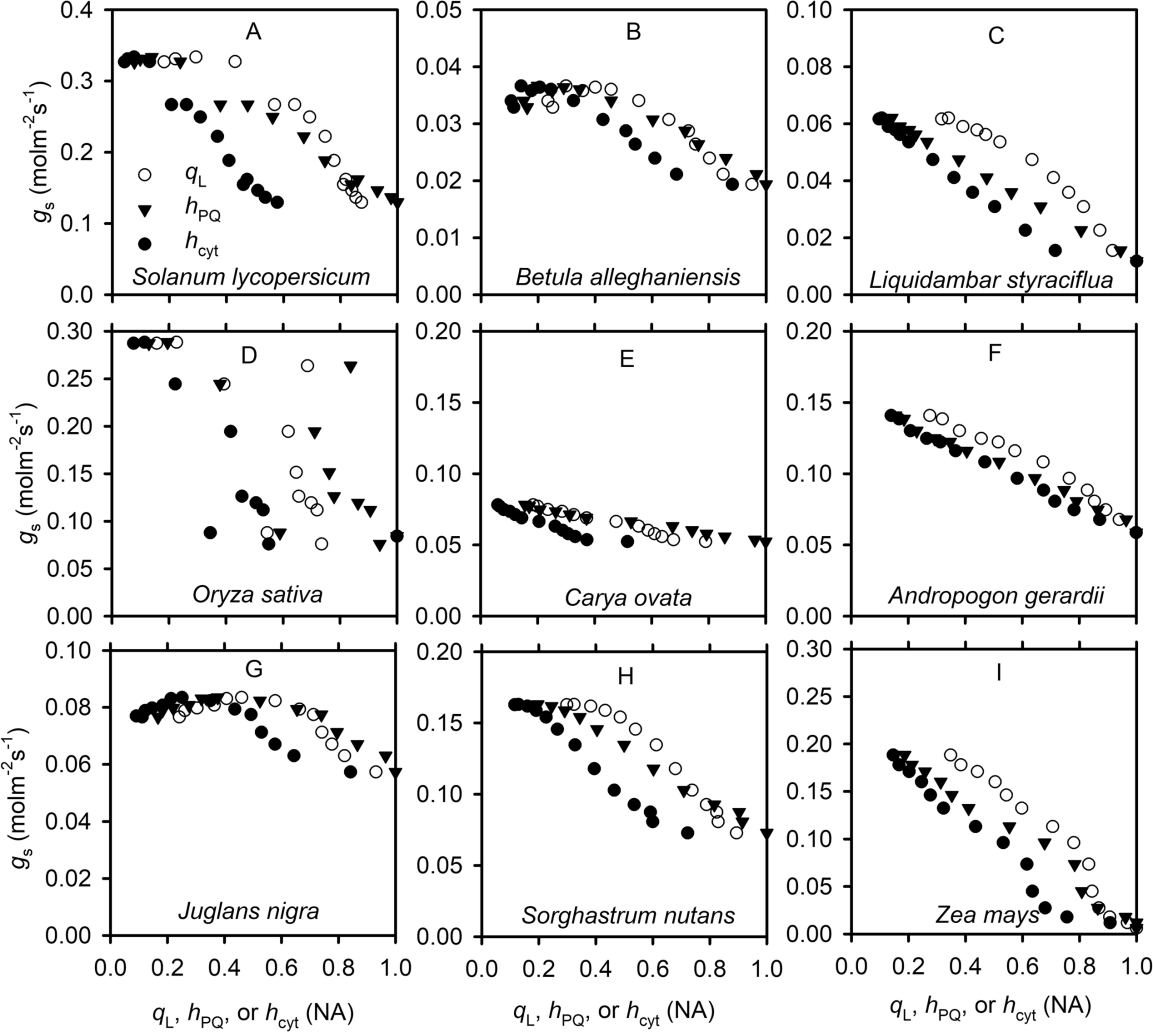
The light‐induced variations of stomatal conductance with the redox states of PSII, plastoquinone, and cytochrome b_6_f complex. The variations in stomatal conductance (*g*_s_), the fraction of open photosystem II reaction centers under the assumption of lake model (*q*_L_, cycle), the oxidized fraction of mobile plastoquinone pool (*h*_PQ_, triangle), and the fraction of cytochrome b_6_f complex available for linear electron transport (*h*_cyt_, solid dot) are from the light response curves of different species under an ambient CO_2_ partial pressure of ~40 Pa.

**FIGURE 8:**
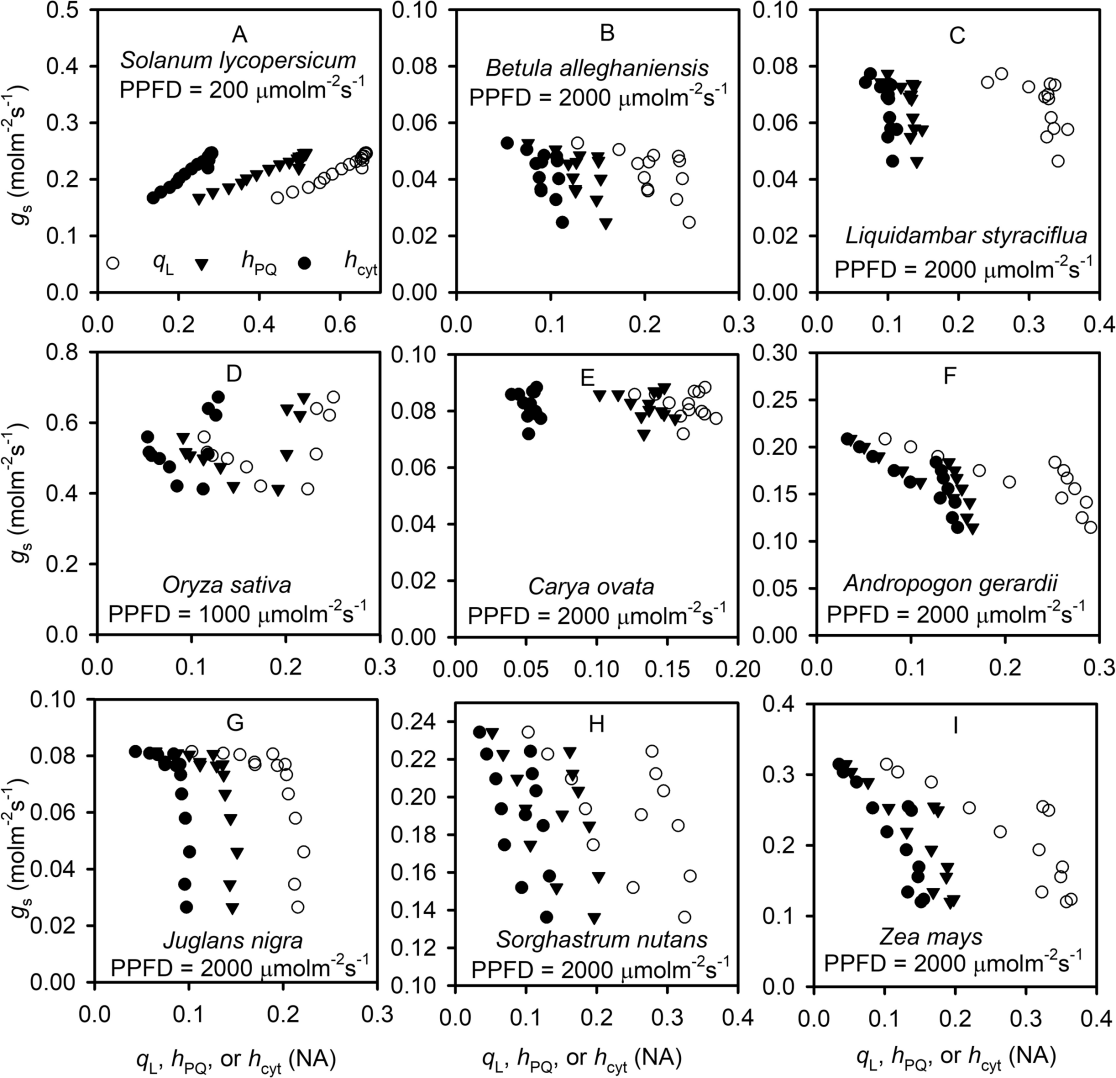
The CO_2_‐induced variations of stomatal conductance with the redox states of PSII, plastoquinone, and cytochrome b_6_f complex. The variations in stomatal conductance (*g*_s_), the fraction of open photosystem II reaction centers under the assumption of lake model (*q*_L_, cycle), the oxidized fraction of mobile plastoquinone pool (*h*_PQ_, triangle), and the fraction of cytochrome b_6_f complex available for linear electron transport (*h*_cyt_, solid dot) are from the CO_2_ response curves of different species under different levels of photosynthetic photon flux density (PPFD).

The maximal stomatal conductances for water vapor diffusion of the species used in our study differ by almost an order of magnitude. However, there are clear commonalities across the species in the variations of redox states of ETC and thylakoid swelling/shrinking with stomatal conductance (Figures 7 and 8). For all species, *g*_s_ generally decreases with increases in *q*_L_, *h*_PQ_, and *h*_cyt_ when these changes are induced by light (Figure 7). For some species (e.g., Figure 7A,B for *Solanum lycopersicum* and *Betula alleghaniensis* , respectively), *g*_s_ appears not sensitive to variations in low values of *q*_L_, *h*_PQ_, and *h*_cyt_ (i.e., when the ETC is more reduced), but its sensitivity increases as the values of *q*_L_, *h*_PQ_, and *h*_cyt_ further increase (i.e., when the ETC is more oxidized). For CO_2_‐induced changes in *g*_s_ and the redox states of ETC, the relationships are more complex (Figure 8). At low light intensity (e.g., Figure 8A for *S. lycopersicum* ), *g*_s_ increases with increases in *q*_L_, *h*_PQ_, and *h*_cyt_. At high light intensity, *g*_s_ decreases with increases in *q*_L_, *h*_PQ_, and *h*_cyt_ (Figure 8B–I). For some species (e.g., Figure 8C for *Liquidambar styraciflua* and Figure 8G for *Juglans nigra* ), *g*_s_ is initially insensitive to variations in *q*_L_, *h*_PQ_, and *h*_cyt_, but as the ETC gets more oxidized with further increased *q*_L_, *h*_PQ_, and *h*_cyt_, *g*_s_ drops sharply. Except for a few cases (e.g., Figure 7E,F for *Carya ovata* and *Andropogon gerardii* , respectively), *g*_s_ varies nonlinearly with *q*_L_, *h*_PQ_, and *h*_cyt_. Although the variations of *g*_s_ with *q*_L_ do not overlap with those with *h*_PQ_ and *h*_cyt_, the overall shapes of these variations are similar. Nevertheless, in some cases, *g*_s_ variation with *h*_PQ_ appears to be slightly more linear than those with *q*_L_ or *h*_cyt_ (e.g., Figure 7C,I).

**FIGURE 9:**
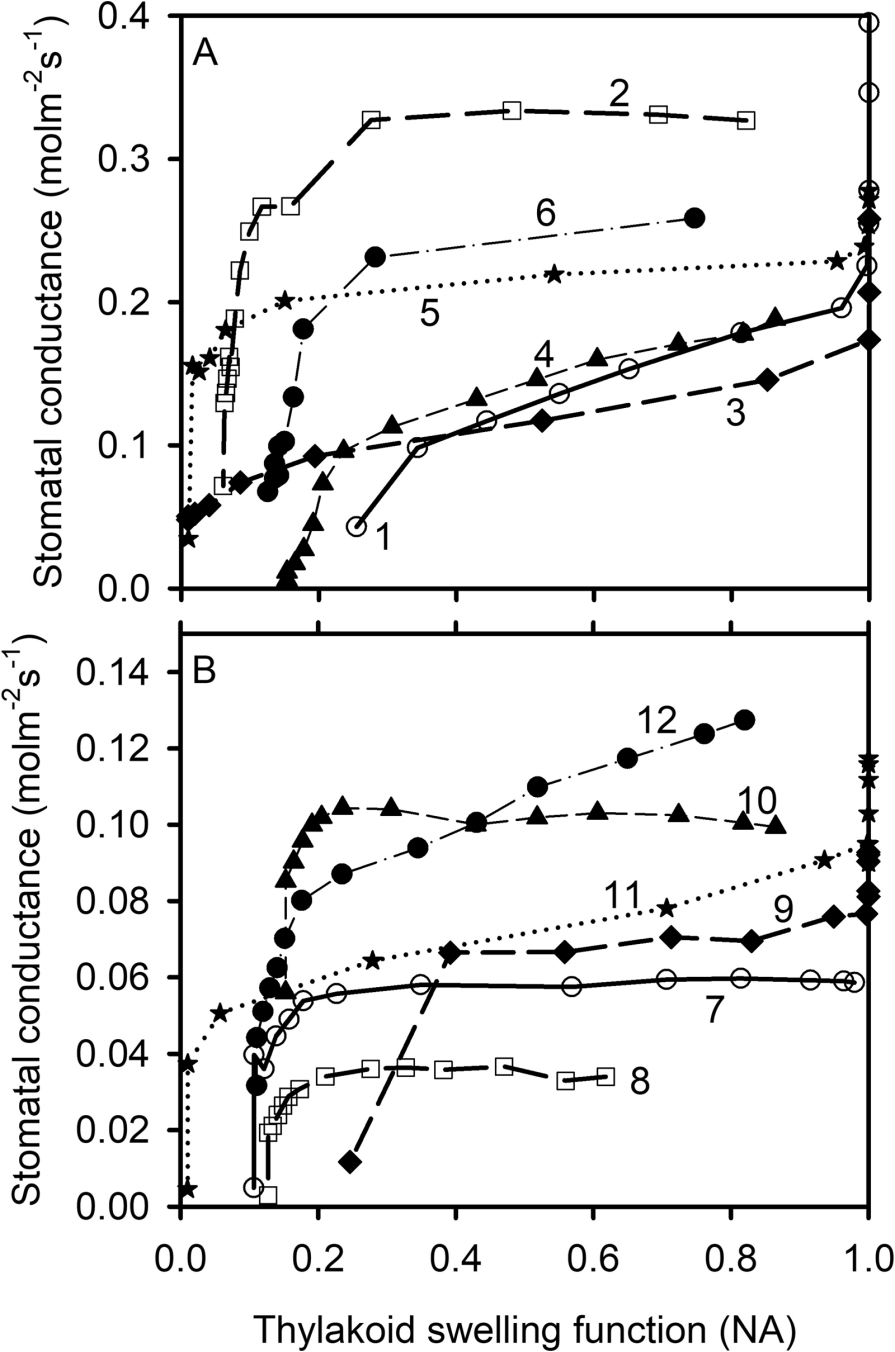
The relationship between stomatal conductance and the thylakoid swelling function. Each curve is marked with a number in A and B and represents the same species/cultivar as shown in Figure 2.

*g*_s_ increases as the thylakoid swells in response to increased light intensity (Figure 9). The increase in *g*_s_ with *f*_s_ is initially steep but becomes much gentler as *f*_s_ further increases. For some species, *g*_s_ even levels off at higher values of *f*_s_ (e.g., *S. lycopersicum* , *C. racemosa* “Ottzam,” and *J. nigra* ).

## 4 Discussion

As the bridge between the photophysical and biochemical reactions, the photochemical reactions play a pivotal role in ensuring the energy supply is balanced with the energy demand. The redox states of ETC reflect how “congested” this bridge is, whereas its “width” is controlled by the swelling/shrinking of thylakoid. This view is supported by our findings that *NPQ* and *g*_s_ covary with the redox states of PSII, PQ, and Cyt and the dynamics of thylakoid ultrastructure. Conceptually, Movie 1 illustrates the essence of our findings.

Although general patterns clearly exist in the relationships of the redox states of ETC, represented by *q*_L_, *h*_PQ_, and *h*_cyt_, and the thylakoid ultrastructural dynamics, quantified by *f*_s_, with *NPQ* and *g*_s_, there are large variations in how these general patterns are expressed for particular species. *NPQ* controls the allocation of the harvested energy between photosynthesis (electron transport) and thermal dissipation. Its multiple components are all carefully regulated by plants to protect chloroplasts and plant cells from deleterious effects of excessive light in rapidly fluctuating light environments (Goss and Lepetit 2015). At moderate to high light levels, more harvested energy is allocated to regulated thermal dissipation than to photosynthesis (photochemical dissipation); however, the energy allocation ratio of non‐photochemical to photochemical quenching varies tremendously across species (Figure S4). This ratio, which increases with light intensity, can be evaluated as the variation in the threshold light intensity at which the energy allocation ratio is equal to 1. Across species, the threshold light intensity varies from less than 300 μmol m^−2^ s^−1^ to over 1000 μmol m^−2^ s^−1^, suggesting that species differ dramatically in energy use strategies. The large variations in *NPQ* across species are determined by plant life histories, growth forms, leaf age, and habitats (Demmig‐Adams et al. 2014). The regulation of *NPQ* is likely a key component of plant energy use strategies. Similar to plant water use strategies, the diverse relationships of *NPQ* with *q*_L_, *h*_PQ_, *h*_cyt_, and *f*_s_ across species may reflect the diversity of plant energy use strategies and therefore that of plant photosynthetic productivity in different environments (Murchie and Ruban 2020). Although plant water use strategies have been studied intensively (Kannenberg et al. 2021), few studies have investigated plant energy use strategies. However, such studies may yield critical insights into the evolution of plants in land environments and potential responses to climate change.

Similarly, there are variations in the sensitivity of stomal conductance to changes in the redox states of PSII, PQ, and Cyt (Figures 7 and 8), particularly for CO_2_ response curves (Figure 8). With respect to its sensitivity to the thylakoid ultrastructural dynamics, the stomatal conductance of some species reaches a plateau when the thylakoid is still swelling, whereas others maximize their stomatal conductance only after the thylakoid is fully swollen (Figure 9). Such differences across species may be related to plant water use strategies (Kannenberg et al. 2021). Plants have a spectrum of water use strategies, ranging from isohydry at one extreme to anisohydry at another. The position of a plant species at this spectrum is subject to stomatal control (Tardieu and Simonneau 1998). As far as we know, plant water use strategies have never been studied in conjunction with the regulation of photosynthetic electron transport in general and the thylakoid dynamics in particular. However, in hindsight, it should be obvious there are tight connections between these critical aspects of plant physiology, given the close coupling between electron transport, carbon assimilation, and transpiration during photosynthesis. Plant water use strategies may be better understood and predicted if they are studied together with the regulation of photosynthetic electron transport and thylakoid swelling/shrinking, and vice versa.

This study focuses on general patterns across species. This focus allows us to draw broadly applicable conclusions for land plants regardless of their specific habitats. The large variations observed across species can be characterized as quantitative rather than qualitative (i.e., no opposing patterns are observed). Although it is beyond the scope of the present study to explain exactly the cause of the observed quantitative variations across species, they are expected and important to study because they reflect species adaptations and fitness in local environments. The predictability of plant functional traits from the environment is generally weak because of physiological equifinality and the impact of evolutionary and ecological scales on trait variations and trait–environment relationships (Anderegg 2023). However, it is well known that sun plants have lower grana stacks, higher NPQ and photochemical efficiency, larger PQ pool, and stronger cyclic electron transport than shade plants (Anderson et al. 2012; Shuang et al. 2022). There are more complications for C_4_ plants because their photosynthesis is conducted with a mesophyll–bundle sheath complex, and grana stacks are abundant in mesophyll cells but rare in bundle sheath cells (Mai et al. 2020). All these considerations suggest that the observed species variations will be challenging to explain but can potentially yield new insights about species–environment interactions.

Our results are consistent with the hypothesis of Busch (2014) that the redox state of PQ plays a role in regulating stomatal response to light (Figure 7). Although the responses of *q*_L_, *h*_PQ_, and *h*_cyt_ to environmental variations do not follow the same trajectories, we suspect that, under natural conditions and from a practical point of view, any of the three could be used as a predictor in a model of *g*_s_, given that the relationships of *g*_s_ with *q*_L_, *h*_PQ_, and *h*_cyt_ are broadly similar. Because *q*_L_ can be monitored with PAM fluorometry whereas *h*_PQ_ and *h*_cyt_ have to be inferred from such measurements with the model of Gu et al. (2023), it is more convenient to use *q*_L_ as a predictor of *g*_s_ than using *h*_PQ_ or *h*_cyt_ as Głowacka et al. (2018) and Kromdijk et al. (2019) already did. However, given the large variations in the *q*_L_–*g*_s_ relationships observed in our study, future studies should investigate whether the form of a *g*_s_ model analogous to that of Ball et al. (1986) and Medlyn et al. (2011) is the most suitable when *q*_L_ is used as a predictor of *g*_s_.

Our present study does not constitute a definitive test of the bellows theory regarding the thylakoid structure and function of land plants proposed by Gu et al. (2022). But our results appear to be consistent with some of the predictions made by that theory. The bellows theory suggests that the thylakoid swelling/shrinking is coordinated with the dynamics of guard cell turgor and stomatal conductance (Movie 1). The coordination is possible as the volumes of both thylakoid and guard cells are controlled by similar osmotic mechanisms caused by ion movements with potassium as the primary osmolyte for guard cells (Fischer 1968) and probably chloride for thylakoid (Li et al. 2021; Li et al. 2020; Beebo et al. 2013). These controls can be synchronized by their shared responses to environmental conditions. The bellows theory posits that the thylakoid ultrastructural dynamics, which have been observed with electron microscopy (Packer et al. 1965; Murakami and Nobel 1967; Krause 1973; Kirchhoff et al. 2011; Kirchhoff 2014; Li et al. 2020), control macromolecular blocking/collision probability, direct diffusional pathlength, division of Cyt function between linear and cyclic electron transport, luminal pH via osmotic water fluxes, and separation of pH dynamics between granal and lamellar lumens in response to environmental variations. These thylakoid ultrastructural controls, in conjunction with the maximal physical separation between PSII in grana stacks and PSI in stromal lamellae (Anderson and Anderson 1980; Danielsson et al. 2004), support the asymmetrical photophysical and photochemical functions and photoprotection priorities of these two photosystems. These controls complement NPQ and biochemical feedbacks to ensure that the supply of electron transport products (e.g., NADPH and ATP) balances the supply of CO_2_ to Rubisco. Under favorable environmental conditions, swollen grana stacks permit free movement of photosynthetic electron carriers, whereas swollen guard cells permit free movement of CO_2_ and water vapor across stomata. Under stress, shrunk grana stacks block movement of electron carriers to prevent photodamage to vulnerable photosystems, particularly PSI whose photodamage is costly and slow to repair as compared to that of PSII (Caffarri et al. 2014), whereas shrunk guard cells close stomata to prevent desiccation (Movie 1). The grana stacks‐based bipartite architecture of thylakoid of land plants provides homeostasis in fluctuating light environments, protects photosystems in drought, and represents an adaptation of photosynthetic machinery to dry and high irradiance conditions to improve fitness of higher plants in challenging land environments.

The bellows theory unifies many well‐known but seemingly disconnected phenomena of thylakoid structure and function of land plants. It suggests that it was by no accident that land plants emerged from within Charophyta (perhaps from terrestrial unicellular charophytes), which happen to have grana stacks. Land environments are much more challenging than aquatic environments for photosynthetic organisms (Gu et al. 2022). Over land, water availability is more volatile and sunlight fluctuates more rapidly. Nitrogen is also more limiting, which not only constrains Rubisco activities but also reduces the potential of nitrate assimilation as a sink for electrons under excessive light conditions. These land‐aquatic differences mean that the energy supply–demand balance is harder to achieve and therefore more robust regulatory mechanisms are needed on land than in water. Having grana stacks, Charophyta may have paved the way for higher plants to successfully conquer the land. The bellows theory explains why grana stacks are ubiquitous in land plants but rare in cyanobacteria and algae. It also explains why PSII is more frequently photodamaged than PSI (Larosa et al. 2018; Alboresi et al. 2019), why shade leaves have taller grana stacks than sun leaves (Anderson et al. 2012), and why higher plants have much smaller capacity for diverting electron flow to oxygen than algae and cyanobacteria do (Badger et al. 2000; Asada 2006; Shirao et al. 2013). The presence of grana stacks and the improved regulatory coordination of photophysical, photochemical, and biochemical reactions may have also made the photoprotective functions of flavodiiron proteins (FLVs) obsolete, explaining their loss from flowering plants. FLVs compete with NADPH for electrons and thus may act as a negative factor for photosynthesis when the ETC is well protected (Alboresi et al. 2019). Of relevance to the present study are the theory's predictions regarding how the thylakoid swelling/shrinking is related to the dynamics of stomatal conductance and NPQ. The bellows theory predicts that as stomata open to allow more CO_2_ and water vapor exchange, thylakoid swells to allow more efficient photosynthetic electron transport (Movie 1); conversely, as stomata close to limit CO_2_ and water vapor exchange, thylakoid shrinks to limit electron transport as a preemptive photoprotection mechanism for PSI, which is functionally more valuable but structurally more vulnerable than PSII. As a result, thylakoid swelling/shrinking is positively correlated with stomatal conductance, allowing electron transport in thylakoid to be balanced with gas exchange through stomata. Figure 9 appears to support these predictions. The bellows theory also predicts that photosynthetic electron transport is regulated by the ultrastructural control before thylakoid is fully swollen. But after thylakoid is fully swollen, the ultrastructural control is lost. Under this condition, the Calvin–Benson cycle is fully activated, allowing NPQ and photosynthetic controls of Cyt activities to serve as the dominant regulations of electron transport. Therefore, the light‐induced thylakoid swelling is correlated with the development of NPQ under non‐stress conditions during the dark‐to‐light transition. Furthermore, as the osmotic water influx that causes thylakoid to swell in light dilutes proton concentration in lumen, thylakoid swelling slows the development of NPQ. However, as thylakoid approaches its full expansion and the osmotic water dilution of luminal proton concentration decreases, NPQ develops more rapidly. These predictions are consistent with the patterns shown in Figures 5 and 6. It is interesting to point out that the relationships between *NPQ* and *q*_L_, *h*_PQ_, and *h*_cyt_ (Figure 3) appear to consist of two distinctive, quasi‐linear segments with the segment for the more reduced ETC (lower values of *q*_L_, *h*_PQ_, and *h*_cyt_) having a steeper slope than the segment for the more oxidized ETC (lower values of *q*_L_, *h*_PQ_, and *h*_cyt_). This is also consistent with the prediction by the bellows theory that the control of electron transport by NPQ differs between before and after the thylakoid is fully swollen.

## 5 Conclusion

Photosynthesis depends on the seamless operation of photophysical, photochemical, and biochemical reactions connected in series. Simultaneous studies of these reactions can yield new insights regarding how different regulatory mechanisms work together to achieve this feat in volatile environments. Our study suggests that the dynamics of redox states of electron transport chain and the thylakoid ultrastructure are closely coupled to the development of non‐photochemical quenching and stomatal conductance to balance dissipation of excessive energy in antenna complexes and electron transport in thylakoid with gas diffusion through stomata and to prevent the overreduction of the electron transport chain. Feedforward mechanisms (i.e., from photophysics to photochemistry to biochemistry/stomatal gas diffusion) play dominant roles in photosynthetic controls, complemented by feedback controls (i.e., from biochemistry/gas diffusion to photochemistry to photophysics). The thylakoid ultrastructural dynamics appear to act as a gatekeeping mechanism for controlling electron transport before Rubisco is activated and stomata are fully open, providing a necessary transition for non‐photochemical quenching to take over the role of controlling electron transport after the lumen is acidified. The results from our study support the hypothesis that the redox state of plastoquinone regulates stomatal conductance and the predictions made by the bellows theory regarding how NPQ and stomatal conductance vary with the light‐induced thylakoid dynamics. Finally, our study suggests that plant water and energy use strategies are intimately linked and should be analyzed together.

## Author Contributions

L.G. planned, designed, and conducted the research and derived all equations. B.G., J.H., T.M., Y.Z., and Y.S. conducted measurements. Y.C.S. checked the derivation of equations and contributed to numerical solutions and discussions. L.G. wrote the first draft and iterated with the coauthors in the subsequent revisions of the manuscript.

## Conflicts of Interest

The authors declare no conflicts of interest.

## Peer Review

The peer review history for this article is available in the Supporting Information for this article.

## Supporting information


**Data S1.** Peer review.

**Figure S1.**Variations of the redox state of the electron transport chain with photosynthetic photon flux density. This figure shows examples of the change in the fraction of open photosystem II reaction centers under the assumption of lake model (*q*_L_, circle), the oxidized fraction of mobile plastoquinone pool (*h*_PQ_, triangle), and the fraction of cytochrome b_6_f complex available for linear electron transport (*h*_cyt_, solid dot) as a function of photosynthetic photon flux density (PPFD). Ambient CO_2_ partial pressure was kept at about 40 Pa. Each plot is for a different species. This figure is reproduced from figure 8 of Gu et al. (2023) with permission granted by Plant, Cell & Environment.
**Figure S2.** Variations of the redox state of the electron transport chain with intercellular CO_2_ partial pressure. This figure shows examples of the change in the fraction of open photosystem II reaction centers under the assumption of lake model (*q*_L_, circle), the oxidized fraction of mobile plastoquinone pool (*h*_PQ_, triangle), and the fraction of cytochrome b_6_f complex available for linear electron transport (*h*_cyt_, solid dot) as a function of intercellular CO_2_ partial pressure (*C*_i_). The photosynthetic photon flux density (PPFD) was 200, 1000, and 1350 μmol m^−2^ s^−1^ for plot A, D, and G, respectively; for all the other plots, PPFD was 2000 μmol m^−2^ s^−1^.
**Figure S3.** The light‐induced redox state relationships between PSII, plastoquinone, and cytochrome b_6_f complex. The oxidized fraction of mobile plastoquinone pool (*h*_PQ_) and the fraction of cytochrome b_6_f complex available for linear electron transport (*h*_cyt_) are plotted against the fraction of open photosystem II reaction centers under the assumption of lake model (*q*_L_) for the light response curves of different species under an ambient CO_2_ partial pressure of ~40 Pa. The 1:1 line is also shown.
**Figure S4.** Variations of energy allocation ratio with photosynthetic photon flux density. The energy allocation ratio is defined as the ratio of non‐photochemical to photochemical quenching. Each curve is marked with a number in A and B and represents a species/cultivar: 1, *Bauhinia glauca*; 2, *Solanum lycopersicum*, tomato Basket Vee; 3 *Solanum lycopersicum*, tomato Growdena; 4, *Zea mays*; 5, *Bauhinia purpurea*; 6, *Oryza sativa*, rice IR64; 7, *Cornus racemosa* “Ottzam”; 8, *Betula alleghaniensis*; 9, *Magnolia henryi*; 10, *Juglans nigra*; 11, *Dichanthelium clandestinum*; 12, *Sorghastrum nutans*.

**Movie 1.** Coordinated diffusions of carbon dioxide (yellow particles) and water vapor (light blue particles) via stomatal pore (left) and electron carriers (blue particles) in the thylakoid (right), according to the bellows theory. The swelling of guard cells facilitates gas exchange between the ambient air and intercellular airspace, whereas the simultaneous swelling of thylakoid facilitates the transport of electrons from photosystem II in grana stacks to photosystem I in stroma lamellae. Osmotic water influxes cause the swelling of both guard cells and thylakoid. For photosynthesis to occur and the safety of the photosynthetic machinery, electron transport must be balanced with gas exchange. Video credit of Nathan Armistead and Jacquelyn DeMink, ORNL, US Dept. of Energy.

## Data Availability

The data used in this study have been made publicly available via www.leafweb.org and tes‐sfa.ornl.gov.
