# Supplementary material for: Regulatory Coordination of Photophysical, Photochemical, and Biochemical Reactions in the Photosynthesis of Land Plants
Source: Plant Direct. 2025 May 26;9(5):e70080. doi: 10.1002/pld3.70080 (PMC12105917; doi:10.1002/pld3.70080)
Supplement: Supplementary file 2 — Figure S1.Variations of the redox state of the electron transport chain with photosynthetic photon flux density. This figure shows examples of the change in the fraction of open photosystem II reaction centers under the assumption of lake model (q L, circle), the oxidized fraction of mobile plastoquinone pool (h PQ, triangle), and the fraction of cytochrome b6f complex available for linear electron transport (h cyt, solid dot) as a function of photosynthetic photon flux density (PPFD). Ambient CO2 partial pressure was kept at about 40 Pa. Each plot is for a different species. This figure is reproduced from figure 8 of Gu et al. (2023) with permission granted by Plant, Cell & Environment. Figure S2. Variations of the redox state of the electron transport chain with intercellular CO2 partial pressure. This figure shows examples of the change in the fraction of open photosystem II reaction centers under the assumption of lake model (q L, circle), the oxidized fraction of mobile plastoquinone pool (h PQ, triangle), and the fraction of cytochrome b6f complex available for linear electron transport (h cyt, solid dot) as a function of intercellular CO2 partial pressure (C i). The photosynthetic photon flux density (PPFD) was 200, 1000, and 1350 μmol m−2 s−1 for plot A, D, and G, respectively; for all the other plots, PPFD was 2000 μmol m−2 s−1. Figure S3. The light‐induced redox state relationships between PSII, plastoquinone, and cytochrome b6f complex. The oxidized fraction of mobile plastoquinone pool (h PQ) and the fraction of cytochrome b6f complex available for linear electron transport (h cyt) are plotted against the fraction of open photosystem II reaction centers under the assumption of lake model (q L) for the light response curves of different species under an ambient CO2 partial pressure of ~40 Pa. The 1:1 line is also shown. Figure S4. Variations of energy allocation ratio with photosynthetic photon flux density. The energy allocation ratio is defined as the r [file PLD3-9-e70080-s001.docx]

**Supplementary Materials**


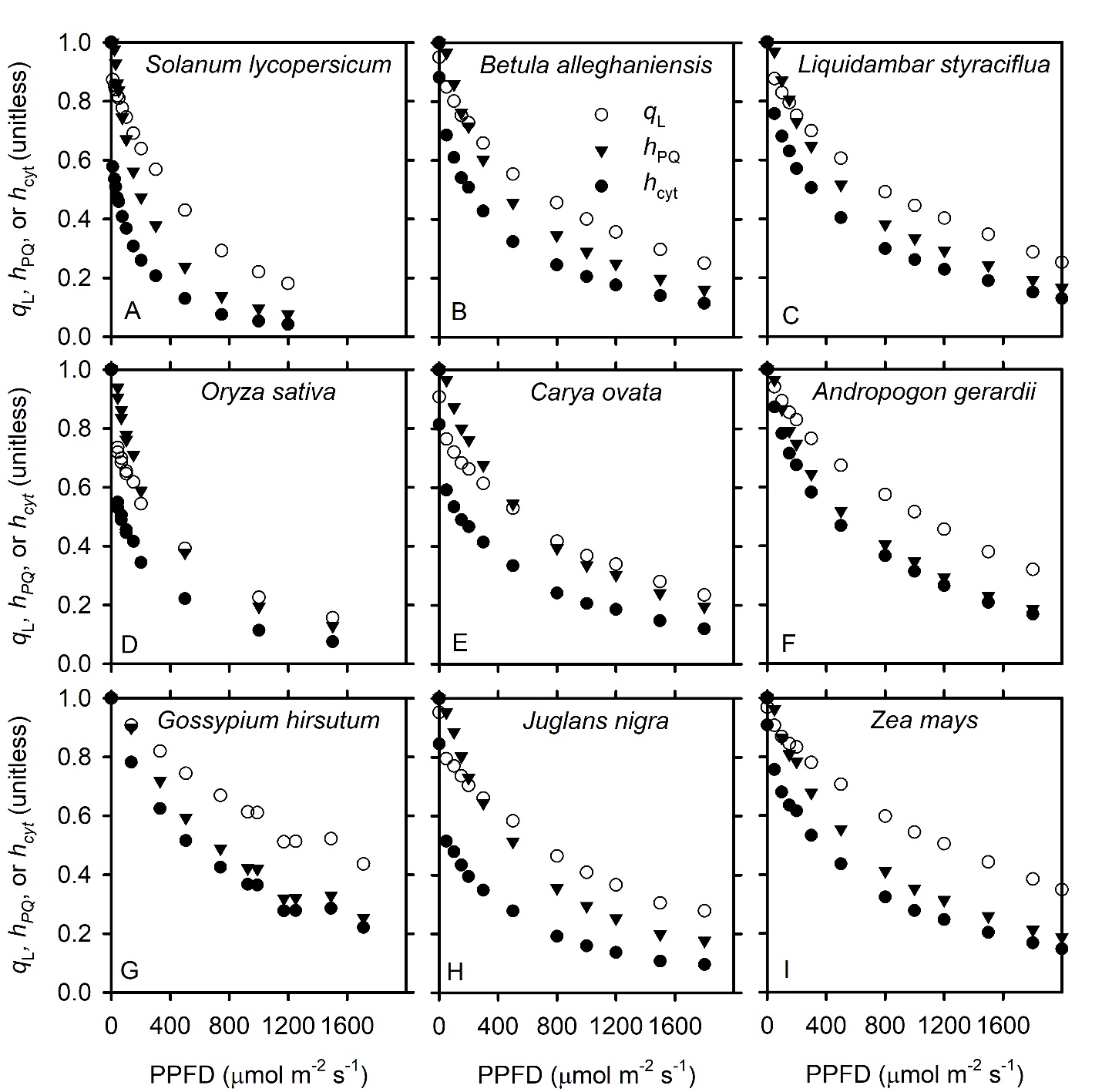


**Supplementary Fig. S1. Variations of the redox state of the electron transport chain with photosynthetic photon flux density.** This figure shows examples of the change in the fraction of open photosystem II reaction centers under the assumption of lake model (*q*_L_, circle), the oxidized fraction of mobile plastoquinone pool (*h*_PQ_, triangle), and the fraction of cytochrome b_6_f complex available for linear electron transport (*h*_cyt_, solid dot) as a function of photosynthetic photon flux density (PPFD). Ambient CO_2_ partial pressure was kept at about 40 Pa. Each plot is for a different species. This figure is reproduced from Fig. 8 of Gu *et al*. (2023a) with permission granted by Plant, Cell & Environment.


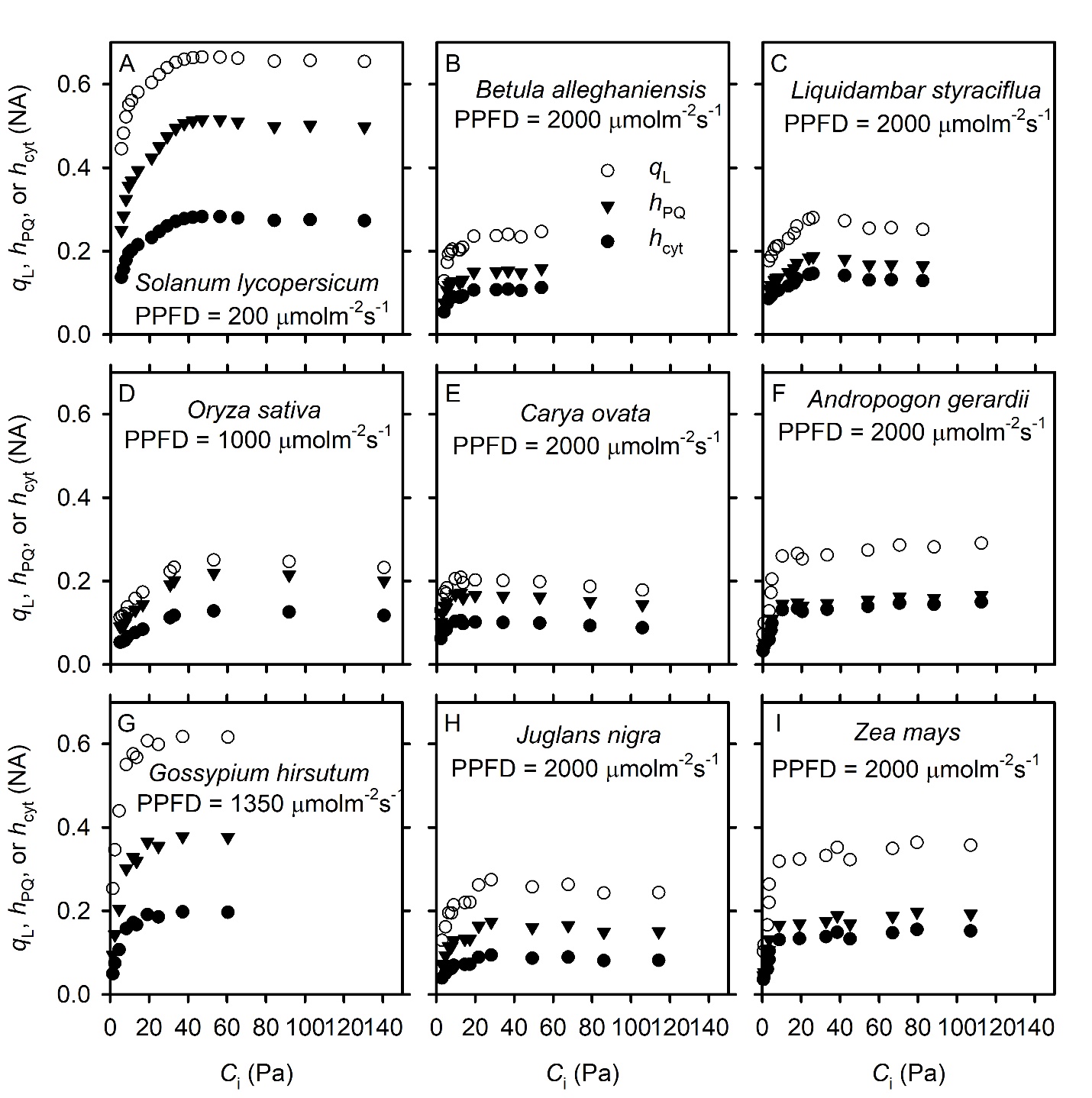


**Supplementary Fig. S2. Variations of the redox state of the electron transport chain with intercellular CO_2_ partial pressure.** This figure shows examples of the change in the fraction of open photosystem II reaction centers under the assumption of lake model (*q*_L_, circle), the oxidized fraction of mobile plastoquinone pool (*h*_PQ_, triangle), and the fraction of cytochrome b_6_f complex available for linear electron transport (*h*_cyt_, solid dot) as a function of intercellular CO_2_ partial pressure (*C*_i_). The photosynthetic photon flux density (PPFD) was 200, 1000, and 1350 µmolm^-2^s^-1^ for plot A, D, and G, respectively; for all the other plots, PPFD was 2000 µmolm^-2^s^-1^.


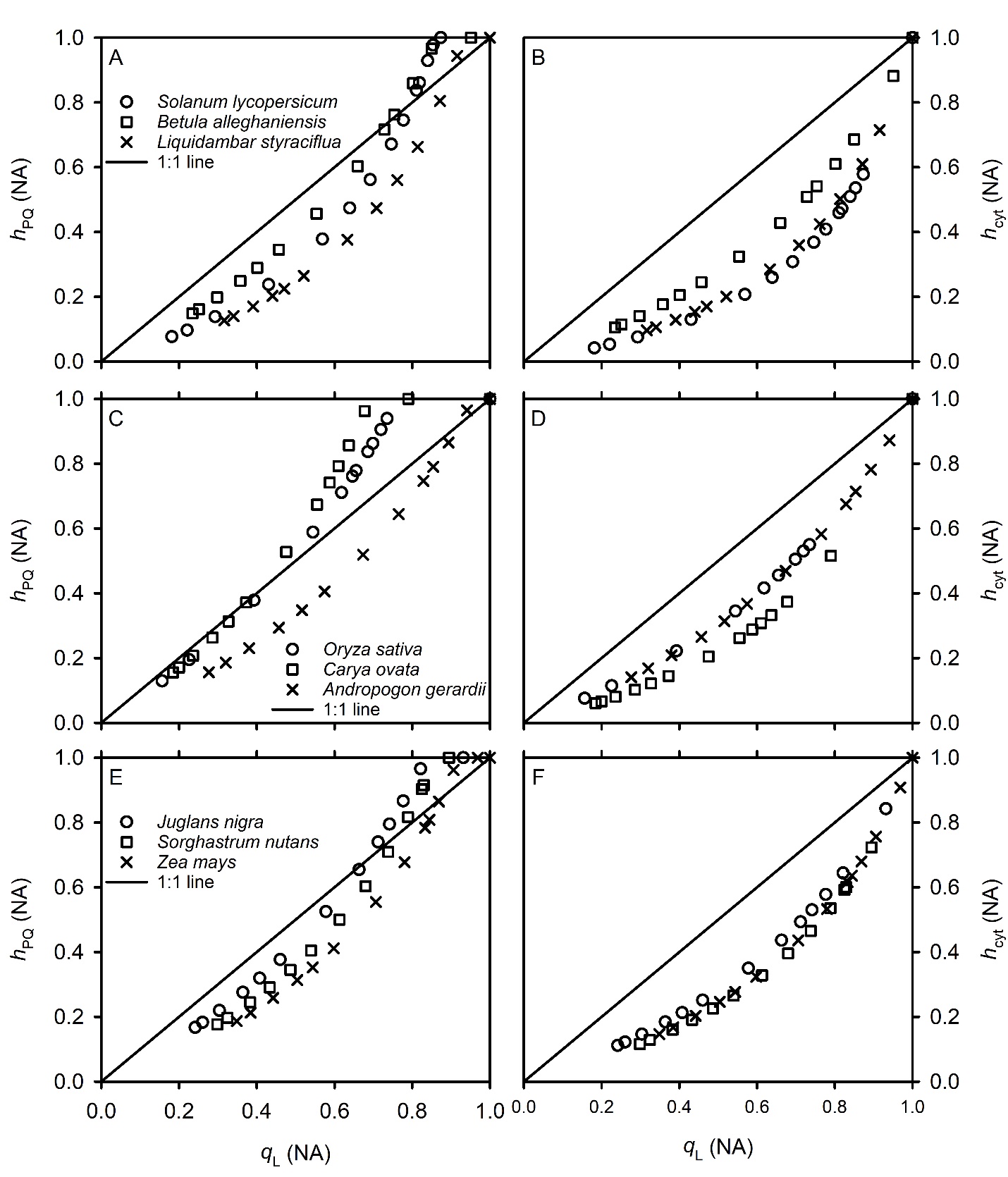


**Supplementary Fig. S3. The light-induced redox state relationships between PSII, plastoquinone, and cytochrome b_6_f complex**. The oxidized fraction of mobile plastoquinone pool (*h*_PQ_) and the fraction of cytochrome b_6_f complex available for linear electron transport (*h*_cyt_) are plotted against the fraction of open photosystem II reaction centers under the assumption of lake model (*q*_L_) for the light response curves of different species under an ambient CO_2_ partial pressure of ~ 40 Pa. The 1:1 line is also shown.


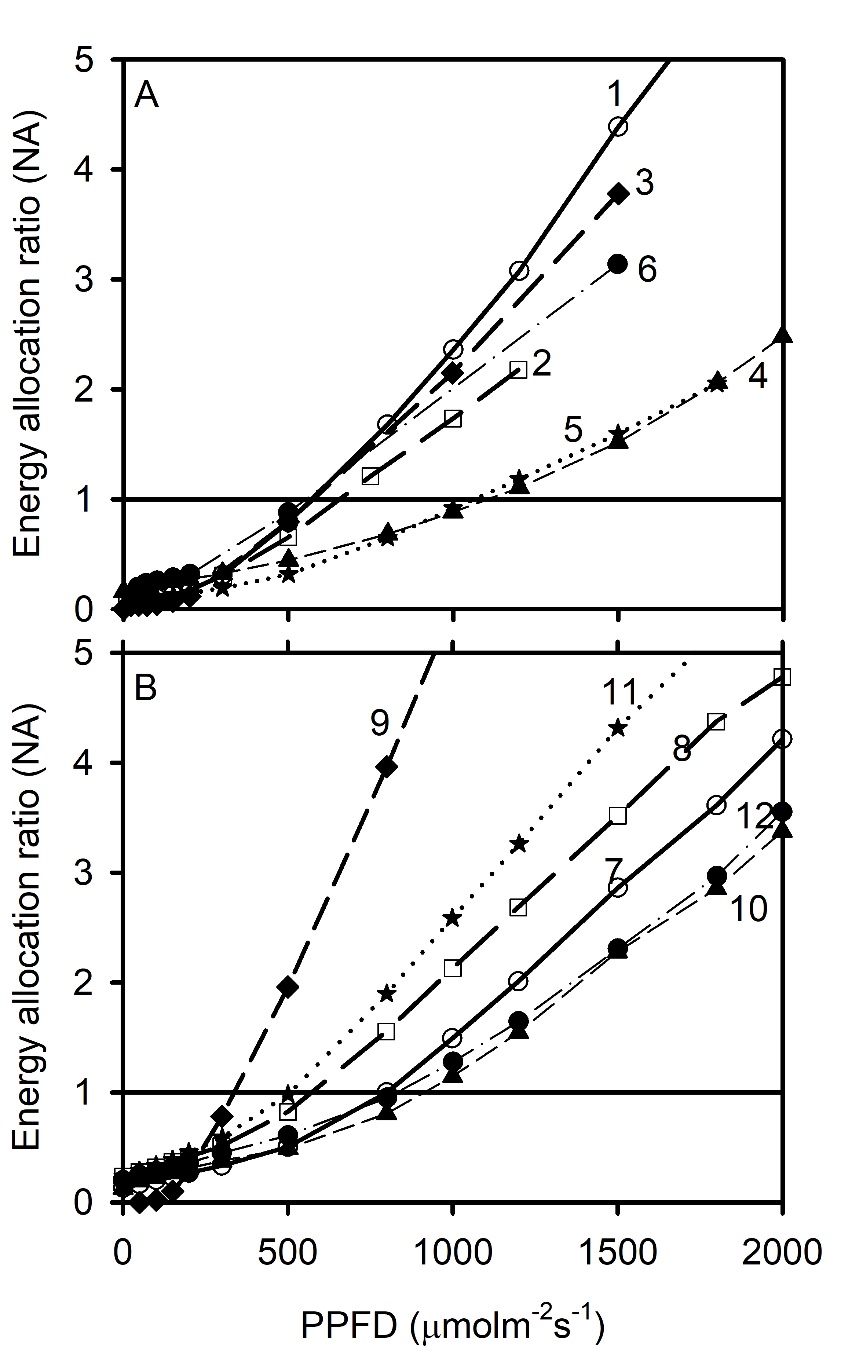


**Supplementary Fig. S4. Variations of energy allocation ratio with photosynthetic photon flux density.** The energy allocation ratio is defined as the ratio of non-photochemical to photochemical quenching. Each curve is marked with a number in A and B and represents a species/cultivar: 1, *Bauhinia glauca*; 2, *Solanum lycopersicum*, tomato Basket Vee; 3 *Solanum lycopersicum*, tomato Growdena; 4, *Zea mays*; 5, *Bauhinia purpurea*; 6, *Oryza sativa*, rice IR64; 7, *Cornus racemosa* ‘Ottzam’; 8, *Betula alleghaniensis*; 9, *Magnolia henryi*; 10, *Juglans nigra*; 11, *Dichanthelium clandestinum*; 12, *Sorghastrum nutans*.

**Move 1**. Coordinated diffusions of carbon dioxide (yellow particles) and water vapor (light blue particles) via stomatal pore (left) and electron carriers (blue particles) in the thylakoid (right), according to the bellows theory. The swelling of guard cells facilitates gas exchange between the ambient air and intercellular airspace whereas the simultaneous swelling of thylakoid facilitates the transport of electrons from photosystem II in grana stacks to photosystem I in stroma lamellae. Osmotic water influxes cause the swelling of both guard cells and thylakoid. For photosynthesis to occur and the safety of the photosynthetic machinery, electron transport must be balanced with gas exchange. Video credit of Nathan Armistead and Jacquelyn DeMink, ORNL, U.S. Dept. of Energy.
