# Supplementary material for: Regulatory Coordination of Photophysical, Photochemical, and Biochemical Reactions in the Photosynthesis of Land Plants
Source: Plant Direct. 2025 May 26;9(5):e70080. doi: 10.1002/pld3.70080 (PMC12105917; doi:10.1002/pld3.70080)
Supplement: Supplementary file 1 — Data S1. Peer review. [file PLD3-9-e70080-s002.pdf]

Plant Direct
Open Access

Regulatory coordination of photophysical, photochemical, and biochemical reactions in the photosynthesis of land plants

Lianhong Gu[1*], Bernard Grodzinski[2], Jimei Han[3,6], Telesphore Marie[2], Yong-Jiang Zhang[4], Yang C. Song[5], Ying Sun[3]

Decision Letter Round 1:

March 2, 2025

Dr. Lianhong Gu
Oak Ridge National Laboratory
Environmental Sciences
Oak Ridge, Tennessee 37831

RE: Regulatory coordination of photophysical, photochemical, and biochemical reactions in the photosynthesis of land plants

Dear Dr. Gu:

Thank you for submitting to Plant Direct. All required reviews have been returned and we have now finished our evaluation of your manuscript. In light of the reviewers' and editor's comments, further revisions are needed before the paper can be accepted for publication in Plant Direct.

Please view the editors' and reviewers' comments below and use their suggestions as a guide while you work on your revision.

When uploading the revised version of this article, please be sure to include the following:

-A word document that contains your response to the reviewers. You should respond to each reviewer comment and note the changes made to the manuscript. If you do not agree with a reviewer's comment and choose not to make a suggested revision, please explain why. Please try to provide as complete an answer as possible to each reviewer's criticisms.
-A tracked changes document with each change highlighted
- A clean version of the latest version of the manuscript

Plant Direct
Open Access

To upload your revision, please click the link below.
https://plantdirect.msubmit.net/cgi-bin/main.plex?el=A2Lr3mm2A1pIQ1I7A9ftdQLjae5UX4t48s4j9jQN9BAZ

In order to provide as timely a service as possible, we ask that your revision is resubmitted within three months after receipt of this request. If an extension is needed, please send a request, along with a brief explanation, to the editorial office at plantdirect@wiley.com .

Please note that, in addition to publishing reviewer comments, the author's responses to review comments will also be published alongside the final version of the paper. If you would not like the author's responses to be published, please contact the editorial office at plantdirect@wiley.com .

Thank you very much for giving us an opportunity to review your work. I look forward to receiving the next version.

Sincerely,

Toshiharu Shikanai

Gustavo MacIntosh

Editor, Plant Direct

-- Editor comments:
One short comment from the editor.
Ls583-585. At least, this would be because of the absence of Flv.

-- Reviewer comments:
Reviewer #1:

In this manuscript, the authors examined relationships between thylakoid ultrastructural dynamics and several measurable parameters of leaves such as redox state of photosynthetic electron transport (PET), non-photochemical quenching (NPQ) and stomatal conductance (gs) using their interesting model. They used the published data, and discussed their data. Some data and their discussion are interesting, but the following major concerns are found.

1) They showed the analyzed results of relationships using nine species' data in Figs. 6-9, but did not discuss the different responses between species. They used the data including C3 and C4 species, and herbaceous/grass and tree species, but did not discuss the physiological significance and underlying mechanisms of the differences. They just showed the divergence. I agree that the thylakoid ultrastructural dynamics may affect the redox state of PET, NPQ and gs if the authors' model is valid, but any significance or underlying mechanisms should be clearly discussed. In L. 515-518, I agree with these sentences, and so the authors should show deepen interpretations of their results.
2) In Yoshida et al. (2010) Plant Cell Physiol 51(5): 836-841, the redox state of PQ is strongly correlated with 1-qp. How can the authors interpret the data by Yoshida et al. (2010)?
3) In Fig. 6B, the authors should use different symbols for different species.

Reviewer #2:

This research investigates how key reactions of photosynthesis work collaboratively to ensure that the energy supply meets the energy demand in different species. This study focused on examining how the redox states of the electron transport chain (ETC), particularly PQ and cyt b6f, and thylakoid swelling/shrinking covary with NPQ and stomatal conductance (gs) across various plant species and climates. The authors adopted a strategy integrating mechanistic model inference with measurements, utilizing a large dataset of joint PAM and gas exchange measurements. They applied the open-closed (OC) photochemical model of photosynthetic electron transport to infer the redox states of PQ and Cyt b6f and a light-induced thylakoid swelling/shrinking function. This study found that NPQ and gs covary with the redox states of the electron transport chain and increase with thylakoid swelling. The findings of this study suggest that plant energy and water use strategies are closely linked by evolution. This study supports the hypothesis that the redox state of plastoquinone regulates stomatal conductance, and the predictions made by the bellows theory regarding thylakoid structure and function in land plants, which proposes that grana stacks act as bellows to regulate electron transport through thylakoid swelling/shrinking.

The reviewer finds the paper very interesting, but thinks that it needs some revisions:
• Theoretical contributions are strong, but additional analyses (e.g., sensitivity testing) and discussions of alternative mechanisms are necessary.
• The Bellows Theory is intriguing but needs empirical validation before it is widely accepted.
• The study's novel insights make it highly valuable, but certain methodological limitations must be addressed before publication.

1. The manuscript effectively identifies a major limitation of prior studies: the lack of an integrated approach to studying photophysical, photochemical, and biochemical reactions of photosynthesis. While previous research gaps are well identified, the introduction could have more explicitly compared this work to previous models to highlight its advancements more clearly.

2. This study introduced a new theoretical framework ("Bellows Theory") suggesting thylakoid ultrastructural control serves as an intermediary regulation before full activation of biochemical and photoprotective mechanisms. Although novel, the Bellows Theory lacks direct experimental confirmation. This could limit its immediate acceptance by the broader scientific community.

3. This study relied heavily on model inference rather than direct measurements of the PQ redox state and thylakoid ultrastructure. This reviewer understands that direct measurement is difficult at this point, so he/she understands that direct measurement is difficult at present and believes that efforts should be made to make the method acceptable to as many scientists as possible. In particular, there is no clear discussion on potential biases in the model inference approach, such as how parameter assumptions might impact the inferred values. Thus, it would be beneficial to include more details on model validation procedures, whether alternative models were tested, and a sensitivity analysis of inferred redox states to the input parameters.

4. The results are well-structured and logically lead to the conclusions of the study. However, the figures are highly data-dense; thus, for general readers, a conceptual diagram summarizing key relationships could improve clarity. Furthermore, some findings, such as species-specific variations in gs sensitivity to the PQ redox state, are not fully explained; thus, more discussion on ecological relevance is needed.

5. In this study, a global optimization algorithm was used to fit the parameters. However, without sensitivity analysis, the robustness of these parameter estimates is unclear. Overfitting

could be an issue, particularly for species-specific responses. The authors should conduct a sensitivity analysis to determine how parameter assumptions influence the model predictions.

6. This study strongly supports the Bellows Theory but does not explore alternative hypotheses that might explain the observed NPQ-thylakoid swelling correlation. Are there other possible mechanisms that could account for these findings? Discuss potential alternative models or mechanisms that could drive the observed relationships.

7. This study spans multiple species, but species-specific differences in NPQ and gs responses to redox states are substantial. The authors state that plant energy and water use strategies are linked but do not deeply analyze how different plant functional groups (e.g., C3 vs. C4 species) may exhibit unique regulatory strategies. Provide more discussion on species-specific responses and whether the findings are universally applicable or more relevant to specific plant types.

8. This study relied heavily on a mechanistic model to infer the redox state of PQ, thylakoid swelling, and other physiological parameters. However, the same model is used to validate key hypotheses, such as the Bellows Theory. This creates the risk of circular reasoning, where inferred data are used to confirm the assumptions of the model itself. To strengthen the study, the authors should clearly acknowledge this limitation in the discussion, consider using independent experimental data (e.g., electron microscopy or spectroscopic techniques) to validate the inferred parameters, and conduct sensitivity analyses to assess how assumptions about model parameters influence the conclusions.

Decision Letter Round 2:

April 24, 2025
Dr. Lianhong Gu
Oak Ridge National Laboratory
Environmental Sciences
Oak Ridge, Tennessee 37831

MSID: 2025-01715R1
MS TITLE: Regulatory coordination of photophysical, photochemical, and biochemical reactions in the photosynthesis of land plants

Plant Direct
Open Access

Dear Dr. Lianhong Gu:

I am pleased to inform you that your manuscript "Regulatory coordination of photophysical, photochemical, and biochemical reactions in the photosynthesis of land plants" has been accepted for publication in Plant Direct.

Your article will appear online in the next available issue of Plant Direct. To ensure your article gets published as quickly as possible, please pay attention to the steps detailed below. We have found that most of the delays happen at this stage, especially at the payment stage, so please respond as quickly as possible when prompted.

License Agreement: Once your article has been accepted it will move to Production and undergo admin and file checking - you may receive an email with any queries we have at this stage. When all required items are received by the publisher and queries resolved, the corresponding author will receive an email from Wiley's Author Services system which will ask them to log on at https://authorservices.wiley.com/bauthor and will present them with the appropriate license for completion. Your article cannot be published until both the signed license agreement and payment of the article fee have been received.

Payment of the Open Access Article Publication Fee: All articles published in Plant Direct are fully open access: immediately and freely available to read, download and share. Plant Direct charges a publication fee to cover the publication costs. The corresponding author for this manuscript should have already received a quote from the payments team (cs-openaccess@wiley.com) with the estimated article publication fee; please email cs-openaccess@wiley.com if this has not been received. The corresponding author should log on to the Wiley Author Services site, where the publication fee can be paid by credit card or an invoice. Pro Forma can also be requested. Payment of the publication charge must be received before the article will be published online.

Proofs: You will have the opportunity to look over your paper once more when you receive the author proofs for your article. The proofs will be with you in approximately two weeks. Please note that, in addition to publishing reviewer comments, the author's responses to review comments will also be published alongside the final version of the paper. If you would not like the author's responses to be published, please contact the editorial office at plantdirect@wiley.com .

Promotion of your article: You can help your research get the attention it deserves! Check out Wiley's free Promotion Guide for best-practice recommendations for promoting your work at www.wileyauthors.com/eeo/guide. And learn more about Wiley Editing Services which offers professional video, design, and writing services to create shareable video abstracts, infographics, conference posters, lay summaries, and research news stories for your research at www.wileyauthors.com/eeo/promotion.

Thank you again for your contribution to Plant Direct. If you have any questions, feel free to contact the editorial office at plantdirect@wiley.com .

Sincerely,

Toshiharu Shikanai

Gustavo MacIntosh

Editor, Plant Direct

-- Editor comments

-- Reviewer comments:
Reviewer #1:

The authors improved the concerns that I raised before. They did not change two comments that I mentioned before, but they appropriately responded to my comments. There is no flaw in the revised version.

Reviewer #2:

I think that the revised version of the manuscript (2025-01715) is well-improved. The authors have responded conscientiously to this reviewer's comments, some of which may have been difficult to address, and I have no additional comments to make.

Plant Direct Open Access

Response to Reviewer:

Response to Comments on

"Regulatory coordination of photophysical, photochemical, and biochemical reactions in the photosynthesis of land plants"

Response to Editor's comments

Comment 1: One short comment from the editor.

Ls583-585. At least, this would be because of the absence of Flv.

Response 1: Thanks for this insightful suggestion! The function of flavodiiron proteins (FLVs) and its loss from angiosperms are indeed very relevant to the discussions of this study. In revising the manuscript, we have added a discussion on how the improved regulatory coordination of photophysical, photochemical, and biochemical reactions in the photosynthesis of flowering plants due to grana stacks may have made the photoprotective function of FLVs obsolete.

Response to Reviewer #1's comments

Overall Comment: In this manuscript, the authors examined relationships between thylakoid ultrastructural dynamics and several measurable parameters of leaves such as redox state of photosynthetic electron transport (PET), non-photochemical quenching (NPQ) and stomatal conductance (gs) using their interesting model. They used the published data, and discussed their data. Some data and their discussion are interesting, but the following major concerns are found.

Overall Response: Thanks for appreciating our research and for providing valuable suggestions. We have revised the manuscript to address in detail the concerns raised by this reviewer.

Comment 1: They showed the analyzed results of relationships using nine species' data in Figs. 6-9, but did not discuss the different responses between species. They used the data including C3 and C4 species, and herbaceous/grass and tree species, but did not discuss the physiological significance and underlying mechanisms of the differences. They just showed the divergence. I agree that the thylakoid ultrastructural dynamics may affect the redox state of PET, NPQ and gs if the authors' model is valid, but any significance or underlying mechanisms should be clearly

discussed. In L. 515-518, I agree with these sentences, and so the authors should show deepen interpretations of their results.

<u>Response 1</u>: We agree with the overall assessment by this reviewer on our manuscript. Our study focuses on general patterns across species. This focus allows us to draw broadly applicable conclusions for land plants regardless of their specific habitats. As shown in this study, species differences are indeed large in their relationships among NPQ, redox states of ETC, and gs. There is no doubt these differences are important as they must play a rule in determining the fitness of species in their respective environments. To explain these differences, we will need to gather detailed information about the environmental conditions (e.g., climate, soil, and hydrology) and potential competitors for each species used in this study. Such studies can yield new insights regarding plant water and energy use strategies as this reviewer agrees (L. 515-518) and should be more fully pursued in separate efforts. The species differences with respect to coordinated regulations of photosynthesis are all quantitative rather than qualitative and don't affect the main conclusions about general patterns across species in our study. The revised manuscript now adds discussions on species differences and their physiological significance.

<u>Comment 2:</u> In Yoshida et al. (2010) Plant Cell Physiol 51(5): 836-841, the redox state of PQ is strongly correlated with 1-qp. How can the authors interpret the data by Yoshida et al. (2010)?

<u>Response 2</u>: Thanks for pointing out the study of Yoshida et al. (2010). This is an interesting paper which we read during the preparation of our manuscript. Its experimental findings based on the HPLC analysis (e.g., their Figure 3) were consistent with our modeling findings (e.g., our Figure 1). But due to our oversight, the finished version of the manuscript did not note the consistency between the two different (experimental – modeling) studies. We apologize for that. The revised manuscript now includes a discussion on Yoshida et al. (2010). Note that our study uses the lake connectivity model (qL) while Yoshida et al. (2010) uses the puddle connectivity model (qP). We have used both the lake and puddle models in our analyses and the results are similar, following our early study (Gu et al. 2023).

Gu L, Grodzinski B, Han J, Marie T, Zhang Y-J, Song YC, Sun Y. 2023. An exploratory steady-state redox model of photosynthetic linear electron transport for use in complete modeling of photosynthesis for broad applications. Plant, Cell and Environment 46: 1540-1561.

<u>Comment 3</u>: In Fig. 6B, the authors should use different symbols for different species.

<u>Response 3</u>: There are just too many points in Fig. 6B. If a different symbol is used for a different symbol, we would run out of common symbols in the graphic tool we use and the plot would be hard to read. As we responded to Comment 1, the focus of the present study are the common

patterns across species and differences among species can be more rigorously pursued in separate studies which will require gathering more information about species habitats.

Response to Reviewer #2's comments

Overall Comment: This research investigates how key reactions of photosynthesis work collaboratively to ensure that the energy supply meets the energy demand in different species. This study focused on examining how the redox states of the electron transport chain (ETC), particularly PQ and cyt b6f, and thylakoid swelling/shrinking covary with NPQ and stomatal conductance (gs) across various plant species and climates. The authors adopted a strategy integrating mechanistic model inference with measurements, utilizing a large dataset of joint PAM and gas exchange measurements. They applied the open-closed (OC) photochemical model of photosynthetic electron transport to infer the redox states of PQ and Cyt b6f and a light-induced thylakoid swelling/shrinking function. This study found that NPQ and gs covary with the redox states of the electron transport chain and increase with thylakoid swelling. The findings of this study suggest that plant energy and water use strategies are closely linked by evolution. This study supports the hypothesis that the redox state of plastoquinone regulates stomatal conductance, and the predictions made by the bellows theory regarding thylakoid structure and function in land plants, which proposes that grana stacks act as bellows to regulate electron transport through thylakoid swelling/shrinking.

The reviewer finds the paper very interesting, but thinks that it needs some revisions:

• Theoretical contributions are strong, but additional analyses (e.g., sensitivity testing) and discussions of alternative mechanisms are necessary.

• The Bellows Theory is intriguing but needs empirical validation before it is widely accepted.

• The study's novel insights make it highly valuable, but certain methodological limitations must be addressed before publication.

Overall Response: Thank you for being positive on our study. This reviewer has identified important areas that can greatly advance integrative studies of photosynthetic regulations promoted by our manuscript. Following the suggestions made by this reviewer, we have revised the manuscript substantially, particularly with respect to the three bullet points stated above.

Comment 1: The manuscript effectively identifies a major limitation of prior studies: the lack of an integrated approach to studying photophysical, photochemical, and biochemical reactions of photosynthesis. While previous research gaps are well identified, the introduction could have

more explicitly compared this work to previous models to highlight its advancements more clearly.

<u>Response</u>: *Suggestion adopted. We have revised the introduction to place the present study in a broader context and identified connections and differences with previous efforts.*

<u>Comment 2:</u> This study introduced a new theoretical framework ("Bellows Theory") suggesting thylakoid ultrastructural control serves as an intermediary regulation before full activation of biochemical and photoprotective mechanisms. Although novel, the Bellows Theory lacks direct experimental confirmation. This could limit its immediate acceptance by the broader scientific community.

<u>Response</u>: *We understand these assessments by the reviewer. Any theory will remain a hypothesis until it is rejected or confirmed by experiments. As in any new development in science, we expect it will be a gradual process for major evidence and as well as acceptance to build up over time. We hope more researchers will analyze our theory against experimental findings. However, there have been plenty of experimental studies with electron microscopy that showed that thylakoids swell in light and shrink in dark (Packer et al. 1965; Murakami and Nobel 1967; Krause 1973; Kirchhoff et al. 2011; Kirchhoff 2014). Also, Reviewer #1 informed us the study by Yoshida et al. (2010) which used the high-performance liquid chromatography (HPLC) method to determine the redox state of plastoquinone in Arabidopsis thaliana. Their findings are consistent with the model-inferred results shown in our Figure 1. Nevertheless, we still need to conduct experiments to monitor simultaneously the dynamics of NPQ, redox states of ETC, thylakoid ultrastructure, and stomatal conductance in responses to systematic variations in living environments. Such experiments will need technological breakthroughs. Currently we are in discussion with scientists at the Spallation Neutron Source (SNS) located at the Oak Ridge National Laboratory (https://neutrons.ornl.gov/sns) about the potential of using neutron scattering imaging to monitor the dynamics of thylakoid in live plants. Please stay tuned.*

Kirchhoff H., Hall C., Wood M., Herbstová M., Tsabari O., Nevo R., Charuvi D., Shimoni E., Reich Z. (2011) Dynamic control of protein diffusion within the granal thylakoid lumen. Proceedings of the National Academy of Sciences of the United States of America, 108, 20248 – 20253.

Kirchhoff H. (2014) Structural changes of the thylakoid membrane network induced by high light stress in plant chloroplasts. Philosophical Transactions of the Royal Society B Biological Sciences, 369, 20130225. http://dx.doi.org/10.1098/rstb.2013.0225.

Krause G.H. (1973) The high-energy state of the thylakoid system as indicated by chlorophyll fluorescence and chloroplast shrinkage. Biochimica et Biophysica Acta (BBA) – Bioenergetics, 292, 715-728.

Murakami S., Nobel P.S. (1967) Lipids and light-dependent swelling of isolated spinach chloroplasts. Plant and Cell Physiology, 8, 657–671.

Packer L., Siegenthaler P., Nobel P.S. (1965) Light-induced volume changes in spinach chloroplasts. Journal of Cell Biology, 26, 593–599.

Yoshida K., Shibata M., Terashima I., Noguchi K. (2010) Simultaneous Determination of In Vivo Plastoquinone and Ubiquinone Redox States by HPLC-Based Analysis. Plant and Cell Physiology, 51, 836–841, https://doi.org/10.1093/pcp/pcq044

Comment 3: This study relied heavily on model inference rather than direct measurements of the PQ redox state and thylakoid ultrastructure. This reviewer understands that direct measurement is difficult at this point, so he/she understands that direct measurement is difficult at present and believes that efforts should be made to make the method acceptable to as many scientists as possible. In particular, there is no clear discussion on potential biases in the model inference approach, such as how parameter assumptions might impact the inferred values. Thus, it would be beneficial to include more details on model validation procedures, whether alternative models were tested, and a sensitivity analysis of inferred redox states to the input parameters.

Response: Although the redox state of plastoquinone can be monitored with the HPLC and the thylakoid structural changes can be observed with electron microscopy, these techniques cannot be applied on live plants to monitor systematic variations in responses to environmental variations. As we mentioned above, we are currently exploring the possibility of using neutron scattering imaging to monitor thylakoid structural dynamics and we hope we will be able to carry out some experiments in the future. The OC model used in the present study was described fully in Gu et al. (2023) which validated the model with the leave-one-out cross-validation approach on about two dozens of species. Sensitivity tests were conducted to assess importance of different process representations (see, for example, Figure 2 vs Figure 4, and Figure 3 in Gu et al. 2023). Gu et al. (2023) compared the OC model with a more complex QAQB model and found that the OC and QAQB models performed almost equally well. But because the QAQB model has more parameters to estimate, it can lead to overfitting and suffer from collinearity. Gu et al. (2023) provides a freely accessible tool (https://onlinelibrary.wiley.com/doi/full/10.1111/pce.14563) for anyone to test the model. The supplementary materials of Gu et al. (2023) also included statistics about parameters estimated. In the revised manuscript, we provide a brief summary of testing results from Gu et al. (2023).

Gu L, Grodzinski B, Han J, Marie T, Zhang Y-J, Song YC, Sun Y. 2023. An exploratory steady-state redox model of photosynthetic linear electron transport for use in complete modeling of photosynthesis for broad applications. Plant, Cell and Environment 46: 1540-1561.

<u>Comment 4</u>: The results are well-structured and logically lead to the conclusions of the study. However, the figures are highly data-dense; thus, for general readers, a conceptual diagram summarizing key relationships could improve clarity. Furthermore, some findings, such as species-specific variations in gs sensitivity to the PQ redox state, are not fully explained; thus, more discussion on ecological relevance is needed.

<u>Response</u>: Not sure if the reviewer had access to the MP4 Video that was submitted in conjunction with the manuscript. The video simulation was created for the exact reason that this reviewer suggests: it serves as a conceptual diagram to show key relationships emphasized in the study. It illustrates the coordinated diffusions of carbon dioxide and water vapor via stomatal pore and electron carriers in the thylakoid. The swelling of guard cells facilitates gas exchange between the ambient air and intercellular airspace whereas the simultaneous swelling of thylakoid facilitates the transport of electrons from photosystem II in grana stacks to photosystem I in stroma lamellae. Osmotic water influxes cause the swelling of both guard cells and thylakoid. For photosynthesis to occur and the safety of the photosynthetic machinery, electron transport must be balanced with gas exchange.

Following the suggestion by this reviewer, we have added a discussion on the ecological relevance of the species-specific variations in gs sensitivity to the PQ redox state.

<u>Comment 5:</u> In this study, a global optimization algorithm was used to fit the parameters. However, without sensitivity analysis, the robustness of these parameter estimates is unclear. Overfitting could be an issue, particularly for species-specific responses. The authors should conduct a sensitivity analysis to determine how parameter assumptions influence the model predictions.

<u>Response</u>: Agreed. Please see Response to Comment 4. Against we emphasize the usefulness of the publicly available tool in Gu et al. (2023).

<u>Comment 6</u>: This study strongly supports the Bellows Theory but does not explore alternative hypotheses that might explain the observed NPQ-thylakoid swelling correlation. Are there other possible mechanisms that could account for these findings? Discuss potential alternative models or mechanisms that could drive the observed relationships.

<u>Response</u>: This is a great question. We obviously cannot rule out alternative hypotheses that may explain the observed NPQ-thylakoid swelling correlation. However, we do know the following:

- *The energy-dependent NPQ is activated by the acidification of the lumen*
- *The acidification of the lumen is due to water splitting and proton translocation from the stroma to lumen as a result of linear and cyclic electron transports*

- *Thylakoid swelling facilitates electron transport whereas thylakoid shrinking hinders electron transport*

Thus, even if alternative hypotheses exist, our hypothesis is the most parsimonious because it does not require us to make additional assumptions. An interesting question that is raised by the editor regards the loss of flavodiiron proteins (FLVs) and their photoprotective functions in flowering plants. It is possible that the improved regulatory coordination of photosynthesis in flowering plants may have made the photoprotective functions of FLVs obsolete. Our hypothesis is the most parsimonious one but it does not mean it is the correct one or even the best one. We are eager to conduct new experiments such as neutral scattering imaging to explore our theory in more detail. We hope the publication of our study will lead to more competitive hypotheses from our colleagues.

Comment 7: This study spans multiple species, but species-specific differences in NPQ and gs responses to redox states are substantial. The authors state that plant energy and water use strategies are linked but do not deeply analyze how different plant functional groups (e.g., C3 vs. C4 species) may exhibit unique regulatory strategies. Provide more discussion on species-specific responses and whether the findings are universally applicable or more relevant to specific plant types.

Response*: Suggestion adopted to some extent. We want to emphasize that the species-specific differences in NPQ and gs responses to redox states are quantitative, not qualitative, i.e., no opposite responses are found across species even though these species are from a wide range of environments. The focus of this study is on general patterns across species. It is not our intention to explain the specific relationships observed for each species in each environment, which would require detailed information on the environments where the species lived and samples were taken. Our findings are broadly applicable in the sense of general patterns, not precise relationships which differ greatly across species. In the revision, we have added discussions on potential physiological causes of differences across species.*

Comment 8: This study relied heavily on a mechanistic model to infer the redox state of PQ, thylakoid swelling, and other physiological parameters. However, the same model is used to validate key hypotheses, such as the Bellows Theory. This creates the risk of circular reasoning, where inferred data are used to confirm the assumptions of the model itself. To strengthen the study, the authors should clearly acknowledge this limitation in the discussion, consider using independent experimental data (e.g., electron microscopy or spectroscopic techniques) to validate the inferred parameters, and conduct sensitivity analyses to assess how assumptions about model parameters influence the conclusions.

<u>Response</u>: While additional independent measurements with electron microscopy, HPLC, and neutron scattering imaging (see our Response to Comment 2) are indeed needed to allow us to make more definitive statements, there is no risk of circularity in our study. The model is just a summary representation of what is known about photosynthetic electron transport along the electron transport chain (See Figure 2 in Gu et al. 2023). Its construct neither requires nor depends on any assumption that needs to be confirmed in the study to be true. For example, the thylakoid dynamic model does not predefine whether thylakoid swelling / shrinking must occur and when it does occur, in which direction (See the sensitivity test results in Figure 3 in Gu et al. 2023). Any relationships that may exist between NPQ, redox states of PQ and cytochrome b6f complex, and gs are never built into the model. Furthermore, the gas exchange and fluorescence measurements are completely independent. Therefore, we can debate whether the reviewer's statement "This creates the risk of circular reasoning, where inferred data are used to confirm the assumptions of the model itself" is justified or not. Nevertheless, the rest of the comment is relevant and we have revised our manuscript accordingly.
